# Supplementary material for: Dendritic Cells or Macrophages? The Microenvironment of Human Clear Cell Renal Cell Carcinoma Imprints a Mosaic Myeloid Subtype Associated with Patient Survival
Source: Cells. 2022 Oct 19;11(20):3289. doi: 10.3390/cells11203289 (PMC9600747; doi:10.3390/cells11203289)
Supplement: Supplementary file 1 [file cells-11-03289-s001.zip › ercDC_cells submission_supplemental information.pdf]

Supplemental information for:

Dendritic cells or macrophages? The microenvironment of human clear cell renal cell carcinoma imprints a mosaic myeloid subtype associated with patient survival

Dorothee Brech[1, $], Anna S. Herbstritt[1,+], Sarah Diederich[1,+], Tobias Straub[2], Evangelos Kokolakis[1], Martin Irmler[3], Johannes Beckers[3,4,5], Florian A. Büttner[6,7], Elke Schaeffeler[6,7], Stefan Winter[6,7], Matthias Schwab[6,7,8,9], Peter J. Nelson[10] and Elfriede Noessner[1,*]

[1] Immunoanalytics/Tissue Control of Immunocytes, Helmholtz Zentrum München, Neuherberg, Germany
[2] Bioinformatics Core Unit, Biomedical Center, Ludwig-Maximilians-University, Munich, Germany
[3] Institute of Experimental Genetics, Helmholtz Zentrum München, Neuherberg, Germany
[4] German Center for Diabetes Research (DZD), Munich, Germany
[5] Chair of Experimental Genetics, Technical University of Munich, Freising, Germany
[6] Dr. Margarete Fischer-Bosch-Institute of Clinical Pharmacology, Stuttgart, Germany
[7] University of Tuebingen, Tuebingen, Germany
[8] Department of Clinical Pharmacology and Department of Pharmacy and Biochemistry, University of Tuebingen, Tuebingen, Germany
[9] German Cancer Consortium (DKTK) and German Cancer Research Center (DKFZ), Heidelberg, Tübingen, Germany
[10] Medizinische Klinik und Poliklinik IV, University of Munich, Munich, Germany
*Correspondence: noessner@helmholtz-muenchen.de.
[$] current affiliation: Bristol-Myers Squibb GmbH & Co. KGaA, Arnulfstraße 29, 80636 Munich, Germany
+ These authors contributed equally to this work.

Supplemental figures and legends

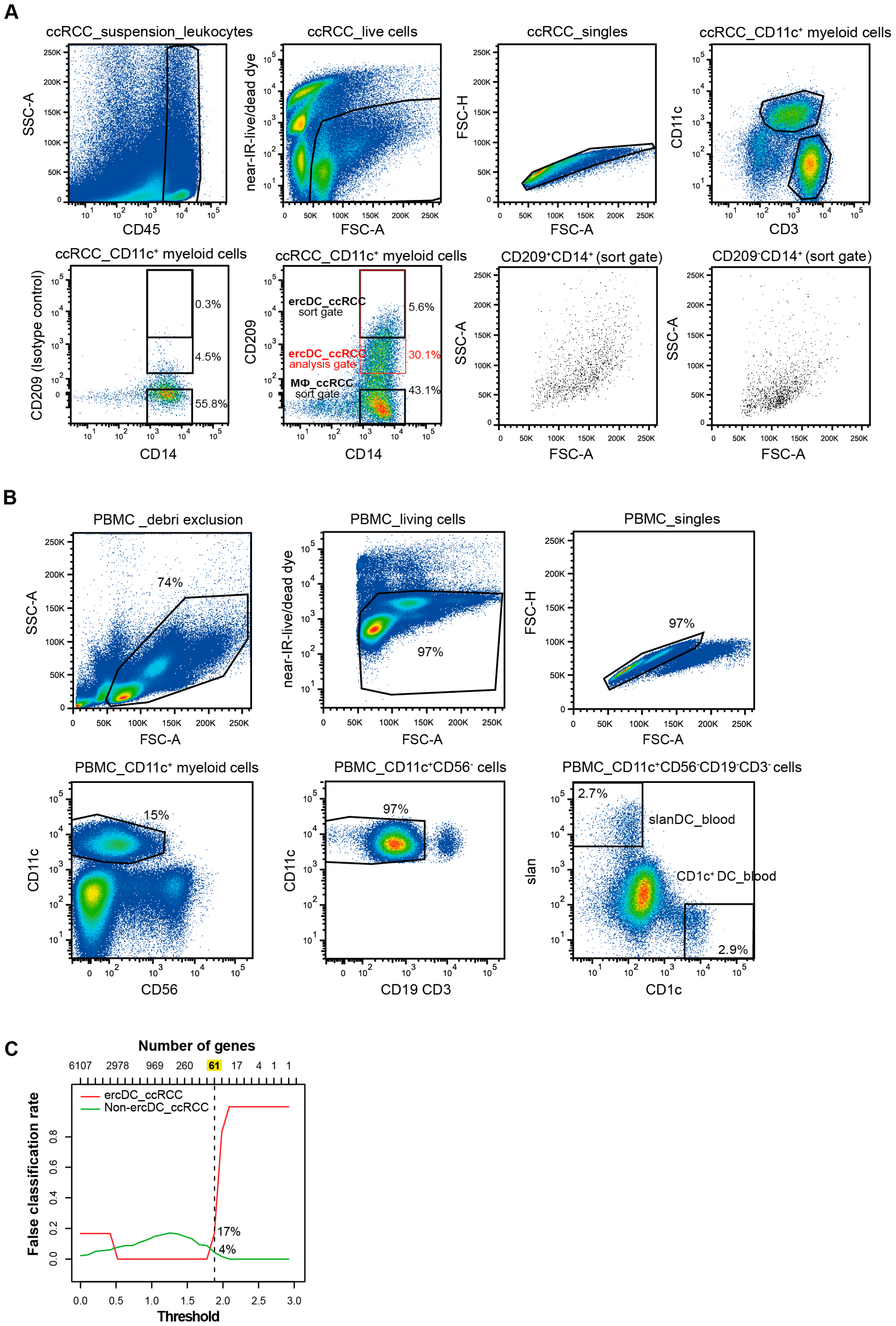

Figure S1: Gating strategy for the sorting of ercDCs and macrophages from ccRCC tissue cell suspension, CD1c$^+$ DCs and slanDCs from PBMCs (blood of healthy donors) and cross-validation of ercDC_ccRCC marker genes. Cross-validation related to figure 4A. (A) ErcDCs and macrophages were sorted from ccRCC tissue-cell-suspensions stained with antibody combination CD45-PeCy7, CD11c-APC, CD3-PB, CD209-PE (all BD Biosciences), CD14-PerCPCy5.5 (eBioscience), LIVE/DEAD® Fixable Near-IR Dead Cell Stain Kit (Thermo Fisher Scientific). (B) Sorting strategy for CD1c$^+$ DC and slanDCs from PBMCs of blood of healthy human donors. CD19/CD56-depleted PBMCs were stained with antibody combination anti-CD11c-PE, anti-CD3-PB (all BD Biosciences), anti-CD56-APC (Beckman Coulter), anti-CD19-PB (Dako), anti-CD1c-PeCy7 (Biolegend), anti-slan-FITC (Miltenyi Biotec) and LIVE/DEAD® Fixable Near-IR Dead Cell Stain Kit (Thermo Fisher Scientific). CD11c+ myeloid cells were selected after gating on CD45$^+$ leukocytes, live and single cells. CD1c$^+$ DCs and slanDCs were sorted from gated CD11c$^+$CD56$^-$CD19$^-$CD3$^-$ cells on the basis of the marker CD1c and slan, respectively. Positivity of both markers was identified with isotype control (not shown). Cell types were selected as following: CD45$^+$ leukocytes were selected in dot plots against SSC, dead cells and doublets were excluded and myeloid cells were selected by CD11c and exclusion of CD3. ErcDCs were selected as CD209 and CD14 co-expressing cells among gated live CD11c$^+$ CD3$^-$ cells. Macrophages within the tissue cell suspension were selected as CD209$^-$CD14$^+$ cells among gated CD45+ live single CD11c$^+$ CD3$^-$ cells. ErcDCs had a larger size (FSC-A) and granularity (SSC-A) than corresponding macrophages. CD209 positivity was judged with the help of an isotype control, CD14 positivity through CD14$^+$ monocytes within PBMC (not shown). Sorts used BD Aria IIIu flow cytometer with 100 µm nozzle and instrument settings modi "purity" or "single cell". (C) Cross-validated misclassification error curves of the nearest shrunken centroid classifier. Depicted are the cross-validation errors for the training set at 30 different threshold levels of shrinkage. The training set comprised 6 ercDC_ccRCC and 182 non-ercDC_ccRCC samples (green line, contains all cell types used in figure 4A). The number of discriminative genes as a function of threshold level in the classifier is indicated on top of the panel. The threshold for defining the set of 61 ercDC_ccRCC marker genes (dashed line) classifier was set in a way that a preferably small group of genes was choosen and the false negative classification rate was below 20 % while the false positive one was below 5%.

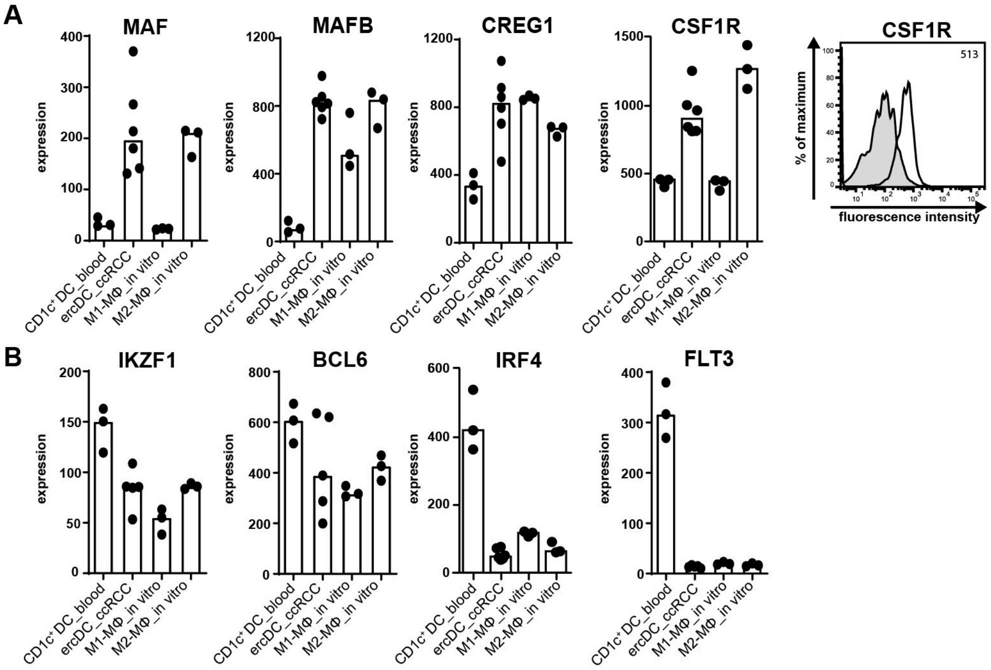

Figure S2: DC- and macrophage-associated genes in ercDCs. (A) Gene expression of macrophage-associated transcription factors and growth factor receptor CSF1R. Bars: median of each group, symbols correspond to individual array replicates of a cell type. Histogram depicts surface expression of CSF1R on ercDCs of ccRCC tissue cell suspensions by flow cytometry (B) Linear expression of genes for DC-associated transcription factors and growth factor receptor FLT3.

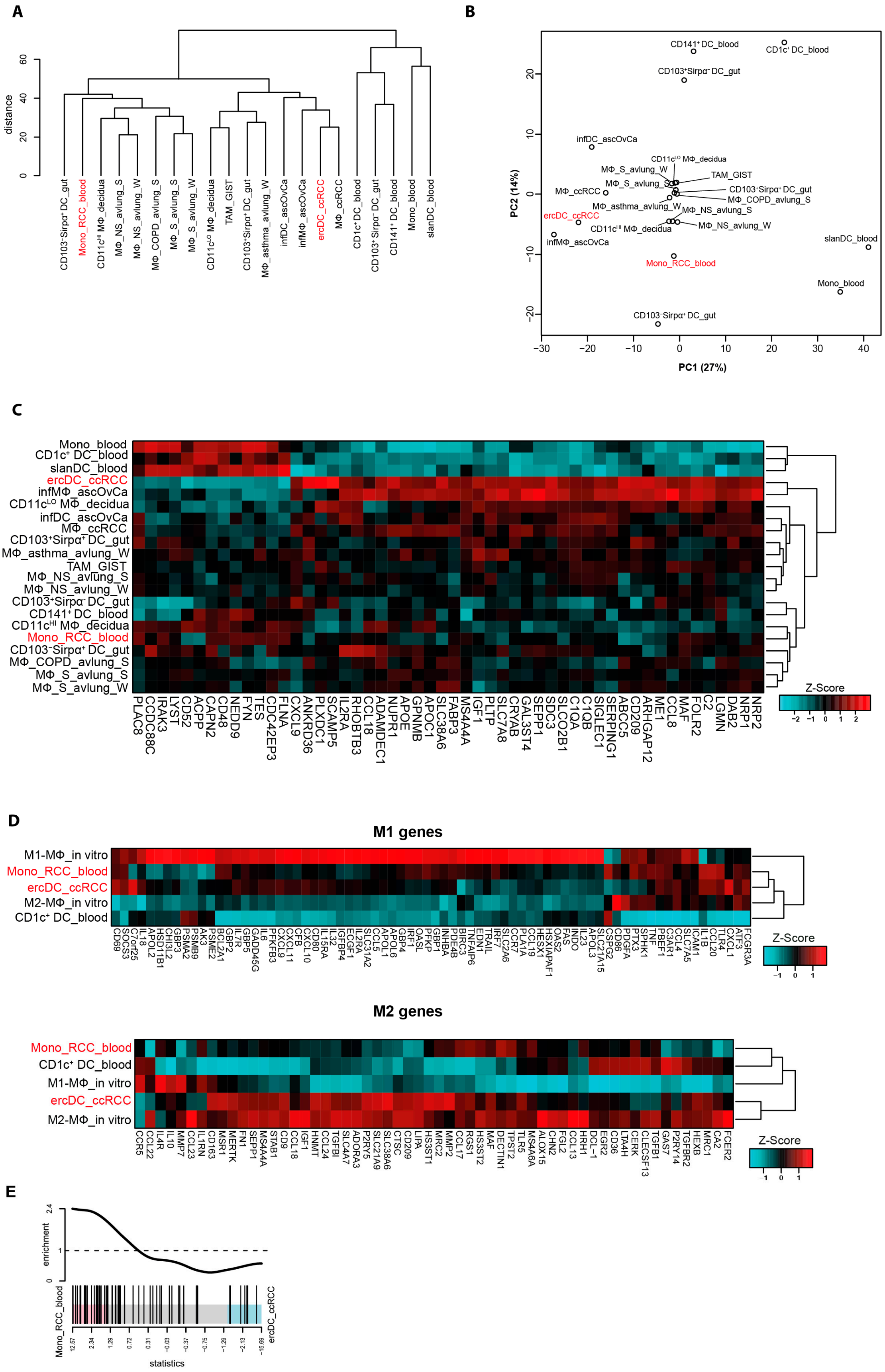

Figure S3: Transcriptome comparison of monocytes from blood of RCC patients with ercDCs from ccRCC tissues and myeloid cells from non-lymphoid tissues after depletion of blood-specific genes. Related to figure 6. Figures are calculated based on expression levels with blood-specific genes excluded. The list of blood-specific genes was from the tissue preferential expressed gene list (blood genes) [86]. (A) Hierachical clustering of indicated cell types based on median expression values of replicate samples considering only "informative" genes (B) PCA on data set shown in (A) depicting the first two PCs that describe 27% and 14% of the variance. (C) Heatmap comparing ercDC_ccRCC marker genes (generated with NSCM [34]) with blood monocytes of RCC patients (Mono_RCC_blood) [85]. Relative expression levels (z-score) of the 61 marker genes are shown. (D) Heatmap showing relative expression levels of genes associated with M1- and M2-macrophages in Mono_RCC_blood and ercDC_ccRCC. (E) Enrichment plot for 73 IL-1R pathway genes defined by merging MSIGDB sets "BIOCARTA_IL1R_PATHWAY", "REACTOME_IL1_SIGNALING",

“PID_IL1_PATHWAY” and “INTERLEUKIN_1_SECRETION”. Genes indicated by vertical bars are spread along the x-axis based on t-statistic comparing ercDC_ccRCC and Mono_RCC_blood cells. Enrichment worm on top shows the relative enrichment of genes in each part of the plot.

Supplemental tables

Table S1: ccRCC tissues. Samples were obtained anonymously with pathologic tumor description but without specification of personal data.

**Patient-ID**	**TNM classification**[a]	**Gender**
RCC-DD1*	n.a.	n.a.
RCC-DD2*	n.a.	n.a.
RCC42	pT3b pN0 G3	Female
RCC66*	pT3a pN0 M1 G3	Male
RCC69	pT3a pN0 (0/4) G2	Female
RCC71	pT3a pN0 G2	Female
RCC74*	pT1a pN0 G1	Male
RCC89*	pT3a pNX G2	Female
RCC90	pT1a pNX G2	Male
RCC91*	pT3a pNX G3	Male
RCC94*	pT1b pN0 G2	Female
RCC97*	pT2b pN0 G2	Female
RCC105	pT3a pNX G3	Male
RCC114	pT3c pN0 G2	Male
RCC118	at least pT3b pN0 G3	Male

[a] Classification according to Union International Contre le Cancer (UICC), T: primary tumor size, N: lymph node involvement, M: distant metastasis; * ccRCC tissues used for gene expression analysis

Table S2A: Myeloid cell types used for Affymetrix arrays (in-house generated data sets).

Cell type	Origin	Name	Marker profile	Replicates	RNA RIN	(Sorted) total cell number
**ercDC**	ccRCC tissue	ercDC_ccRCC	$CD11c^+$ $CD209^+$ $CD14^+$	RCC66	8.6	2.1×10^4
				RCC74	6.1	1.7×10^4
				RCC89	8.0	9.5×10^4
				RCC91	8.0	1.1×10^4
				RCC94	6.7	1.1×10^4
				RCC-DD2	5.6	0.7×10^4
**MΦ**	ccRCC tissue	MΦ_ccRCC	$CD11c^+$ $CD209^-$ $CD14^+$	RCC66	8.8	6.0×10^4
				RCC89	8.0	2.2×10^5
				RCC91	7.3	4.2×10^4
				RCC94	6.1	2.5×10^4
				RCC-DD1	7.8	0.5×10^4
				RCC-DD2	7.8	6.1×10^4
**$CD1c^+$ DC**	Blood healthy	$CD1c^+$ DC_blood	$CD11c^+$ $CD1c^+CD19^-$	Donor 1	9.0	2.0×10^4
				Donor 2	7.4	1.2×10^4
				Donor 3	6.2	1.0×10^4
**slanDC**	Blood healthy	slanDC_blood	$CD11c^+slan^+$ $CD19^-$	Donor 1	7.6	1.5×10^4
				Donor 2	8.8	5.0×10^4
				Donor 3	8.6	2.7×10^4
**Monocytes**[a]	Blood healthy	Mono_blood	$CD14^+$	Donor Pool 1[+]	8.0	5.0×10^5
				Donor Pool 2[+]	8.6	5.0×10^5
				Donor Pool 3[+]	7.9	5.0×10^5
**M1-MΦ**[b]	In vitro healthy	M1-MΦ_in vitro	$CD14^+$	Donor Pool 1[+]	8.2	5.0×10^5
				Donor Pool 2[+]	8.5	5.0×10^5
				Donor Pool 3[+]	8.7	5.0×10^5
**M2-MΦ**[#]	In vitro healthy	M2-MΦ_in vitro	$CD14^+$	Donor Pool 1[+]	8.7	5.0×10^5
				Donor Pool 2[+]	8.8	5.0×10^5
				Donor Pool 3[+]	8.8	5.0×10^5

[a] Monocytes isolated from PBMC using $CD14^+$ microbeads (Miltenyi). [b] M1- and M2-macrophages (MΦ) generated as described [32]. [+] Each pool consists of cells from 5 different donors.

Table S2B: human myeloid cell subtypes of external data sets used for comparison to ercDC_ccRCC.

Name	Origin	No. of replicates	Accession code
$CD141^+$ DC_blood	Blood healthy	3	E-TABM-34 [74]
$CD11c^{HI}$ MΦ_decidua	decidua first trimester	8	GSE22342 [31]
$CD11c^{LO}$ MΦ_decidua		8	
$CD103^+Sirp\alpha^-$ DC_gut	gut	3	GSE50380 [68]
$CD103^+Sirp\alpha^+$ DC_gut		5	
$CD103^-Sirp\alpha^+$ DC_gut		3	
MΦ_NS_avlung_S	avlung	24	GSE13896 [69] (S)
MΦ_S_avlung_S		34	
MΦ_COPD_avlung_S		12	
MΦ_NS_avlung_W	avlung	15	GSE2125 [70] (W)
MΦ_S_avlung_W		15	
MΦ_asthma_avlung_W		15	
TAM_GIST	GIST	12	GSE51697 [71]
infDC_ascOvCa	ascOvCa	5	GSE40484 [73]
Mono_RCC_blood	Blood of RCC	4	GSE38424 [3]

_S_ = smoker, _NS_ = non-smoker, _S = Shaykhiev data; _W = Woodruff data; GIST = gastrointestinal stromal tumor, COPD = chronic obstructive pulmonary disease, av = alveolar; ascOvCa = ascites of ovarian cancer

Table S3: Antibodies. Antibody concentrations for FACS sorting were adjusted to the total cell number of each sample. Isotypes were adjusted to the concentration of respective specific antibody.

Antibody	Label	Species/Isotype	Clon	Company
B7-DC/PD-L2	APC	Mouse IgG1, κ	MIH18	BD
B7-H1/PD-L1	FITC	Mouse IgG1, κ	M1H1	BD
B7-H3	APC	Mouse IgG2b, κ	FM276	Miltenyi Biotec
CD1c	FITC	Mouse IgG2a	AD5-8E7	Miltenyi Biotec
CD1c	PE-Cy7	Mouse IgG1, κ	L161	Biolegend
CD11c	APC	Mouse IgG1	B-ly6	BD
CD11c	A700	Mouse IgG1	B-ly6	BD
CD11c	PE	Mouse IgG1, κ	B-ly6	BD
CD13/ANPEP	APC	Mouse IgG1, κ	WM15	BD
CD14	PB	Maus IgG2a, κ	M5E2	BD
CD14	PerCP-Cy5.5	Mouse IgG1, κ	61D3	eBioscience
CD19	PB	Mouse IgG1, κ	HD37	Dako
CD204/MSR1	FITC	rec. human IgG1	REA460	Miltenyi Biotec
CD206/MRC1	FITC	Mouse IgG1, κ	15-2	Biolgend
CD209	APC	Mouse IgG2b, κ	DCN46	BD
CD209	PE	Mouse IgG2b, κ	DCN46	BD
CD3	PB	Mouse IgG1, κ	UCHT1	BD
CD3	V500	Mouse IgG1	UCHT1	BD
CD32A	unlabeled	Goat IgG	Polyclonal	R&D Systems
CD36	FITC	Mouse IgG2a	FA6-152	Immunotech
CD45	PE-Cy7	Mouse IgG1, κ	HI30	BD
CD45	APC Cy7	Mouse IgG1, κ	HI30	BD
CD56	V450	Mouse IgG1	B159	BD
CD64	FITC	Mouse IgG1, κ	22	Beckman Coulter
CD64	PerCP Cy5.5	Mouse IgG1	10.1	BioLegend
CD68	FITC	Mouse IgG	Y1/82A	BD
CD163	PerCP Cy5.5	Mouse IgG1, κ	GHI/61	BioLegend
CD9	FITC	Mouse IgG1	MEM-61	EuroBioSciences
CSF1R	A700	Mouse IgG1	61708	R&D Systems
FLT3	APC	Mouse IgG1	BV10A4H2	eBioscience
GPNMB	A700	Mouse IgG2b	303822	Novus Biologicals
Isotype	A700	Mouse IgG1, κ	11711	R&D Systems
Isotype	APC	Mouse IgG1, κ	11711	R&D Systems
Isotype	APC	Mouse IgG2b, κ	27-35	BD
Isotype	FITC	Mouse IgG1, κ	MOPC21	BD
Isotype	PE	Mouse IgG1	MOPC21	BD
Isotype	PE	Mouse IgG2b, κ	MPC-11	BioLegend
Isotype	PE-Cy7	Mouse IgG1, κ	MOPC21	BD
Isotype	FITC	Mouse IgM	IS5-20C4	Miltenyi Biotec
MerTK	APC	Mouse IgG1, κ	125518	R&D Sytems
NRP1	FITC	Mouse IgG1	AD5-17F6	Miltenyi Biotec
Slan/M-DC8	FITC	Mouse IgM	RCC-DD-1	Miltenyi Biotec
TIM-3	FITC	Rat IgG2a	344823	R&D Systems
VSIG4	unlabled	Rabbit	polyclonal	Abcam
Anti-goat	A488	Donkey IgG	polyclonal	Thermo Fisher Scientific

Table S4: Patient characteristics of TCGA and Rostock cohort

**TCGA cohort** (n=442)			
**Parameter**		**N, value**	**%**
Sex	Male	287	65
	Female	155	35
Age (year)	Median (range)	60 (29-90)	
Pathologic_T	T1	224	51
	T2	55	12
	T3	158	36
	T4	5	1
Pathologic_N	N0	204	46
	N1	10	2
	NX	228	52
Pathologic_M	M0	353	80
	M1	64	14
	MX	23	5
G	G1	9	2
	G2	188	43
	G3	177	40
	G4	65	15
	GX	1	0.0
	NA	2	0.0
Follow-up time (year)	Median (range)	3.6 (0.0 – 12.4)	
Cancer-specific survival	Cancer-related death	87	20
	Alive/non-cancer-related death	355	80

**Rostock cohort [44,45]**								
**Patient ID**	**Tumor type**	**Age/ gender**	**Tumor size (cm)**	**Tumor stage (tumor-node-metastasis)**	**Nuclear grade, 3-tiered WHO**	**Follow-up (mo)**	**Outcome Status**	**Normal tissue available (NZK)**
**Primary tumor G1**								
12	ccRCC	72/female	3.0	pT2N0 M0V0R0	G1	148	ANED*	X
113	ccRCC	59/male	4.0	pT1 N0M0V0R0	G1	133	ANED	X
158	ccRCC	72/male	3.2	pT2 N0M0V0R0	G1	126	AWD	X
34	ccRCC	65/male	5.2	pT2N0M0V0R0	G1	144	ANED	X
348	ccRCC	59/male	8.0	pT2N0M0V0R0	G1	67	ANED	X
365	ccRCC	62/female	5.5	pT2N0M0V0R0	G1	65	ANED	X
165	ccRCC	77/male	4.5	pT1N0M0V0R0	G1	125	ANED	X
198	ccRCC	70/male	3.8	pT1N0M0V0R0	G1	97	DBOR	X
262	ccRCC	71/female	5.0	pT1N0M0V0R0	G1	96	ANED	
269	ccRCC	53/female	8.0	pT3aN0M0V0R0	G1	94	ANED	
286	ccRCC	65/male	5.5	pT1N0M0V0R1	G1	93	ANED	
288	ccRCC	70/female	5.5	pT1N0M0V0R0	G1	90	ANED	
290	ccRCC	56/male	3.5	pT1N0M0V0R0	G1	89	ANED	
294	ccRCC	62/female	3.0	pT1N0M0V0R0	G1	76	DBOR	
**Primary tumor G3**								
89	ccRCC	64/male	11.0	pT3bN2M1V1R0	G3	2	DOTD	
188	ccRCC	59/male	9.0	pT2 N0M1V0R0	G3	30	DOTD	X
197	ccRCC	63/male	13.0	pT3bN1M1V1R0	G3	23	DOTD	X
254	ccRCC	62/male	12.0	pT3bN0M1V1R0	G3	29	DOTD	
382	ccRCC	56/male	8.0	pT3bN0M1V1R0	G3	60	AWD	X
351	ccRCC	66/female	10.5	pT3bN1M1V1R0	G3	11	DOTD	X
168	ccRCC	58/male	12.0	pT3aN0M0V1R1	G3	46	DOTD	
190	ccRCC	56/male	8.6	pT3bN0M0V1R0	G3	31	DOTD	X

230	ccRCC	55/female	4.0	pT3bN0M0V1R1	G3	15	DOTD	
345	ccRCC	59/male	13.0	pT3aNXM1V0R0	G3	60	AWD	
399	ccRCC	65/female	7.5	pT3aN0M1V0R0	G3	15	DOTD	
510	ccRCC	79/female	5.0	pT1aN0M1V1R0	G3	3	AWD	
283	ccRCC	48/male	5.2	pT1N0M1V1R0	G3	94	AWD	
390	ccRCC	59/female	16.0	pT3bN0M1V1R1	G3	2	DOTD	X

* ANED = alive with no evidence of disease, AWD = alive with disease, DOTD = death of tumor disease, DBOR = death of other reason

Table S5: M1-associated genes [31,32,69]. Bold: ercDC_ccRCC marker gene, purple: ercDC_ccRCC DEGs

Gene name	Gene symbol/alternative name	Informative
**Membrane receptors**		
Complement component 3a receptor 1	C3AR1	yes
CCR7	CCR7	yes
CD69	CD69	yes
CD80	CD80	yes
CD86	CD86	no
Fc fragment of IgG, high affinity Ia, receptor (CD64)	CD64/FCGR1A	yes
Fc fragment of IgG, high affinity IIa, receptor (CD32)	CD32/FCGR2A	yes
Fc fragment of IgG, high affinity IIIa, receptor (CD16)	CD16/FCGR3A	yes
ICAM1	ICAM1	yes
Interleukin 15 receptor α chain	IL15RA	yes
Interleukin 2 receptor α chain	**IL2RA**	yes
Interleukin 7 receptor	IL7R	yes
Toll-like receptor 2	TLR2	yes
Toll-like receptor 4	TLR4	yes
**Cytokines and chemokines**		
CXCL11	CXCL11	yes
CCL14	CCL14	no
CCL15	CCL15	no
CCL19	CCL19	yes
CCL20	CCL20	yes
CCL4	CCL4	yes
CCL5	CCL5	yes
CXCL1	CXCL1	yes
CXCL10	CXCL10	yes
CXCL9	**CXCL9**	yes
Thymidine phosphorylase	TYMP	yes
Interleukin 12B	IL12B	no
Interleukin 15	IL15	no
Interleukin 18	IL18	yes
Interleukin 1B	IL1B	yes
Interleukin 23	IL23	yes
Interleukin 32	IL32	yes
Interleukin 6	IL6	yes
Nicotinamide phosphoribosyltransferase	NAMPT/PBEF1	yes
Tumor necrosis factor ligand superfamily, member 2	TNF	yes
Tumor necrosis factor, α-induced protein 6	TNFAIP6	yes
Tumor necrosis factor (ligand) superfamily, member 10	TRAIL	yes
**Apoptosis associated genes**		
BCL2-related protein A1	BCL2A1	yes
Baculoviral 1AP repeat-containing 3	BIRC3	yes
Tumor necrosis factor receptor superfamily, member 6	FAS	yes
Growth arrest and DNA-damage-inducible, γ	GADD45G	yes
XIAP associated factor-1	HSXIAPAF1	yes
**Transport proteins**		
Solute carrier family 21, member 15	SLC21A15	yes

Solute carrier family 2, member 6	SLC2A6	yes
Solute carrier family 31, member 2	SLC31A2	yes
Solute carrier family 7, member 5	SLC7A5	yes
**Enzymes and other proteins**		
Adenylate kinase 3	AK3	yes
B factor, properdin (complement factor B)	CFB	yes
Chitinase 3-like 2	CHI3L2	yes
Hydroxysteroid (11-β) dehydrogenase 1	HSD11B1	yes
Indoleamine 2,3 dioxygenase 1	IDO1	yes
Nitric oxide synthase 2A (inducible)	NOS2A	no
2′-5′-oligoadenylate synthetase 2	OAS2	yes
2′-5′-oligoadenylate synthetase-like	OASL	yes
Phosphodiesterase 4B, cAMP-specific	PDE4B	yes
6-phosphofructo-2-kinase/fructo-2,6-biphosphatase 3	PFKFB3	yes
Phosphofructokinase	PFKP	yes
Phospholipase A1 member A	PLA1A	yes
Proteasome subunit α type 2	PSMA2	yes
Proteasome subunit β type 9	PSMB9	yes
Proteasome activator subunit 2	PSME2	yes
Sphingosine kinase 1	SPHK1	yes
**Extracellular mediators**		
Apolipoprotein L1	APOL1	yes
Apolipoprotein L2	APOL2	yes
Apolipoprotein L3	APOL3	yes
Apolipoprotein L6	APOL6	yes
Chondroitin sulfate proteoglycan 2	CSPG2	yes
Endothelin 1	EDN1	yes
Insulin-like growth factor binding protein 4	IGFBP4	yes
Inhibin β A	INHBA	yes
Platelet-derived growth factor α	PDGFA	yes
Pentraxin 3	PTX3	yes
**DNA binding factors**		
Activating transcription factor 3	ATF3	yes
Homeobox expressed in ES cells 1	HESX1	yes
Interferon regulatory factor 1	IRF1	yes
Interferon regulatory factor 7	IRF7	yes
Storkhead box 1	STOX1	no
**Signaling-associated genes**		
Guanylate-binding protein 1, IFN-inducible	GBP1	yes
Guanylate-binding protein 2, IFN-inducible	GBP2	yes
Guanylate-binding protein 3	GBP3	yes
Guanylate-binding protein 4	GBP4	yes
Guanylate-binding protein 5	GBP5	yes
Immunoresponsive 1 homolog	IRG1	no
Suppressor of cytokine signaling 3	SOCS3	yes
**Function not known**		
Chromosome 7 open reading frame 25	C7orf25	yes

Table S6: M2-associated genes [31,32,69]. Bold: ercDC_ccRCC marker gene, purple: ercDC_ccRCC signature genes

Gene name	Gene symbol/alternative name	Informative

**Membrane receptors**		
Adenosine A3 receptor	ADORA3	yes
CCR5	CCR5	yes
CD163	CD163	yes
CD36	CD36	yes
CD9	CD9	yes
C-type lectin family 7A	CLEC7A/DECTIN1	yes
C-type lectin domain family 10, member A	CLEC10A/CLECSF13	yes
CXCR4	CXCR4	yes
CD302	CD302/DCL-1	yes
CD209	**CD209/DC-SIGN**	yes
Fc fragment IgE, low affinity II, receptor for (CD23)	FCER2/CD23	yes
G protein-coupled receptor 86	GPR86	yes
Histamine receptor H1	HRH1	yes
IL4R	IL4R	yes
c-mer protooncogene tyrosine kinase	MERTK	yes
Mannose receptor, C type 1	CD206/MRC1	yes
Mannose receptor, C type 2	MRC2	yes
Membrane-spanning 4-domains, subfamily A, member 4A	**MS4A4A**	yes
Membrane-spanning 4-domains, subfamily A, member 6A	MS4A6A	yes
Macrophage scavenger receptor 1	CD204/MSR1	yes
Purinergic receptor P2Y, G-protein coupled, 14	P2RY14	yes
Purinergic receptor P2Y, G protein-coupled, 5	P2RY5/LPAR6	yes
Stabilin 1	STAB1	yes
Transforming growth factor β receptor II	TGFBR2	yes
Toll-like receptor 5	TLR5	yes
**Cytokines and chemokines**		
CCL13	CCL13	yes
CCL17	CCL17	yes
CCL18	**CCL18**	yes
CCL22	CCL22	yes
CCL23	CCL23	yes
CCL24	CCL24	yes
Insulin-like growth factor 1	**IGF1**	yes
Interleukin 10	IL10	yes
IL1 receptor antagonist	IL1RN	yes
TGFB1	TGFB1	yes
**Transport proteins**		
Solute carrier family 21, member 15	**SLCO2B1/SLC21A9**	yes
Solute carrier family 38, member 6	**SLC38A6**	yes
Solute carrier family 4, member 7	SLC4A7	yes
**Enzymes and other proteins**		
Adenosine kinase	ADK	yes
Arachidonate 15-lipoxygenase	ALOX15	yes
Arginase 1	ARG1	no
Carbonic anhydrase II	CA2	yes
Ceramide kinase	CERK	yes
Collagen, type VI, α 2	COL6A2	no
Cathepsin C	CTSC	yes
Hexosaminidase B	HEXB	yes
Histamine *N*-methyltransferase	HNMT	yes
Heparan sulfate (glucosamine) 3-*O*-sulfotransferase 1	HS3ST1	yes
Heparan sulfate (glucosamine) 3-*O*-sulfotransferase 2	HS3ST2	yes
Lipase A cholesterol esterase	LIPA	yes
Leukotriene A4 hydrolase	LTA4H	yes
Matrixmetallopeptidase 2 (gelatinase A, 72-kDa gelatinase)	MMP2	yes
MMP7 (matrilysin, uterine)	MMP7	yes
MMP9 (gelatinase B, 92-kDa gelatinase)	MMP9	yes
Tyrosylprotein sulfotransferase 2	TPST2	yes
**Extracellular mediators**		
Chimerin 2	CHN2	yes
Fibrinogen-like 2	FGL2	yes
Fibrionectin 1	FN1	yes
Selenoprotein P, plasma, 1	**SEPP1**	yes

Transforming growth factor, beta-induced, 68 kDa	TGFBI	yes
**DNA binding factors**		
Early growth response 2	EGR2	yes
Growth arrest-specific 7	GAS7	yes
v-maf musculoaponeurotic fibrosarcoma oncogene homolog	**MAF**	yes
**Signaling associated genes**		
Regulator of G protein signaling 1	RGS1	yes

Table S7: Markers of 3 tissue macrophage subtypes. Adopted from [10], supplemented with genes from [56] (green).

Marker	Gene symbol/ alternative name
**Classical activated, bactericidal macrophages**	
CCL15	CCL15[a]
CCL20	CCL20[b]
CXCL10	CXCL10
CXCL11	CXCL11
CXCL9	CXCL9
IL-12	IL12A/B[b]
iNOS	NOS2[b]
**Wound healing associated, tissue modulating macrophages**	
CCL17	CCL17
CCL18	CCL18
CCL22	CCL22
DCIR	DCIR/CLEC4A
Factor XIII-A	F13A1
IGF-1	IGF1
IL-27Rα	IL27RA
RELMα	FIZZ1
Stabilin 1	STAB1
YM1	YM1
BEX3	NGFRAP1
C1q, subunit A	C1QA
C1q, subunit B	C1QB
COL1A2	COL1A2
COL3A1	COL3A1
COL4A2	COL4A2
COL6A3	COL6A3
FN1	FN1
Gas6	GAS6
HIF-2α	EPAS1
Hsp27	HSPB1
ITGB5	ITGB5
MMP-9	MMP9
PADI4	PADI4
Protein S	PROS1
S100-A4	S100A4
SDC2	SDC2
Serpin F1	SERPINF1
VCAN	VCAN
**Immunoregulatory macrophages**	
CCL1	CCL1
IL-10	IL10
LIGHT	LIGHT/TNFSF14
SPHK1	SPHK1
α2M	A2M
CD9	CD9
c-Maf	MAF
DAB2	DAB2
CD209	CD209/DC-SIGN
DPEP2	DPEP2
IRAK3	IRAK3
CD206	CD206/MRC1
PGDS	PGDS
TNFRSF21	TNFRSF21
TREM2	TREM2
VSIG4	VSIG4

[a] Gene not present in study group and could not be analyzed; [b] Gene not shown in figure 2A because it was only marginally expressed by all cell types or not suitable because it was not strongly expressed by positive controls compared to negative controls

Table S8: Genes associated with invasion and angiogenesis [57], gene set "angiogenesis" from GSEA MSigDB database [39] and factors listed at http://www.sabiosciences.com/rt_pcr_product/HTML/PAHS-024A.html. (as of May 2014)

Gene	Entrez ID
EGF	1950
IL18	3606
IL8	3576
TGFB2	7042
VEGFA	7422
CCL2	6347
CXCL2	2920
CXCL3	2921
CXCL5	6374
FGF1	2246
FGF2	2247
HGF	3082
HIF1A	3091
IGF1	3479
IL15	3600
IL1B	3553
IL6	3569
KDR	3791
MMP2	4313
MMP9	4318
NRP1	8829
NRP2	8828
PDGFA	5154
PGF	5228
PLAU	5328
PTGS1	5742
TEK	7010
TGFA	7039
TGFB1	7040
TGFBR1	7046
TNF	7124
VEGFC	7424
CTSB	1508
ANGPT1	284
FLT1	2321
IL17A	3605
SPP1	6696
GPNMB	10457
MT1G	4495
MMP7	4316
CCL8	6355
CCL22	6367
CCL7	6354
CCL4L2	388372
APOE	348
APOC1	341
TNFAIP6	7130
IL1A	3552
A2M	2
NR1H3	10062
CTSL1	1514
MT1E	4493
PLTP	5360
CCL4L1	9560
ADAMDEC1	27299
DFNA5	1687
MT1H	4496

SLC39A8	64116
FPR3	2359
CCL20	6364
CXCL1	2919
MT2A	4502
SLCO2B1	11309
ACP5	54
TM4SF1	4071
TGM2	7052
NDP	4693
PMP22	5376
ABCA1	19
TM7SF4	81501
LPL	4023
INDO	3620
CCL3	6348
GJB2	2706
CYP27B1	1594
SLC1A3	6507
PTGES	9536
CCL3L1	6349
MAPK13	5603
CCR7	1236
IL23A	51561
VSIG4	11326
STEAP3	55240
SCG5	6447
CCND1	595
CA12	771
WBP5	51186
LGMN	5641
BCAT1	586
EMP1	2012
MAP1LC3A	84557
FBP1	2203
F3	2152
RASGRP3	25780
GSN	2934
CRABP2	1382
ETV5	2119
PPAP2B	8613
IRAK2	3656
CD9	928
SCD	6319

Table S9: List of 61 marker genes of ercDC_ccRCC. Genes were computed with the nearest shrunken centroids method (NSCM) [34]. 39 genes were upregulated, 22 genes downregulated compared to the other cells listed in heatmap figure 4A. Depicted are the shrunken centroid values (positive for upregulated, negative for downregulated genes) that indicate the degree of contribution of the gene to the profile.

Gene symbol	Entrez ID	Shrunken Centroid value
CD209	30835	0.292
IGF1	3479	0.259
PLXDC1	57125	0.200
SEPP1	6414	0.194
FOLR2	2350	0.185
PLTP	5360	0.168
SCAMP5	192683	0.160
NUPR1	26471	0.142
C2	717	0.141
ME1	4199	0.140
GPNMB	10457	0.127
SDC3	9672	0.121
IL2RA	3559	0.100
SLCO2B1	11309	0.099
NRP1	8829	0.088
CCL8	6355	0.086
APOC1	341	0.083
CCL18	6362	0.080
GAL3ST4	79690	0.073
SIGLEC1	6614	0.073
SLC38A6	145389	0.071
C1QA	712	0.051
CRYAB	1410	0.050
SERPING1	710	0.048
ARHGAP12	94134	0.042
APOE	348	0.040
ANKRD36	375248	0.038
DAB2	1601	0.031
RHOBTB3	22836	0.030
ADAMDEC1	27299	0.026
MS4A4A	51338	0.024
NRP2	8828	0.023
ABCC5	10057	0.020
C1QB	713	0.018
LGMN	5641	0.016
FABP3	2170	0.014
CXCL9	4283	0.011
SLC7A8	23428	0.008
MAF	4094	0.003
TES	26136	-0.006
LYST	1130	-0.013
NEDD9	4739	-0.018
CD48	962	-0.023
FYN	2534	-0.025
ACPP	55	-0.033
FLNA	2316	-0.037
IRAK3	11213	-0.045
CAPN2	824	-0.056
LILRA2	11027	-0.058
CYTIP	9595	-0.067
EMR3	84658	-0.071
CDC42EP3	10602	-0.078
LILRA1	11024	-0.084
PLAC8	51316	-0.088
FAM65B	9750	-0.123
CCDC88C	440193	-0.154
S100A12	6283	-0.157
CD52	1043	-0.228
FGR	2268	-0.245
CFP	5199	-0.282
FCN1	2219	-0.461

Table S10: Upregulated ercDC_ccRCC DEGs. Please find list in separate excel file

Table S11: Downregulated ercDC_ccRCC DEGs. Please find list in separate excel file

Table S12: Top 20 overrepresented signaling pathways of upregulated ercDC_ccRCC DEGs in InnateDB analysis [36-38] **(**signaling pathways in orange are significantly overexpressed, adjusted p-value <0.05) or Top 20 overrepresented signaling pathways of ercDC_ccRCC&infMP_ascOvCa in GSEA analysis

**InnateDB upregulated signaling pathway s of ercDC_RCC DEGs**	**Adjusted p-value**
Lysosome	3.99×10^{-7}
Complement and coagulation cascades	1.57×10^{-4}
Beta1 integrin cell surface interactions	7.68×10^{-4}
Collagen biosynthesis and modifying enzymes	0.009
Beta3 integrin cell surface interactions	0.016
HDL-mediated lipid transport	0.020
Phagosome	0.041
ECM-receptor interaction	0.041
Regulation of complement cascade	0.068
NOD-like receptor signaling pathway	0.079
Staphylococcus aureus infection	0.098
Validated transcriptional targets of AP1 family members Fra1 and Fra2	0.113
Integrin cell surface interactions	0.114
Inhibition of matrix metalloproteinases	0.148
Toll-like receptor signaling pathway	0.150
Chemokine receptors bind chemokines	0.158
Degradation of collagen	0.165
Fibrinolysis pathway	0.166
Metal ion SLC transporters	0.166
Platelet amyloid precursor protein pathway	0.166

**GSEA overrepresented signaling pathways of ercDC_ccRCC&infMP_ascOvCa vs crtl group**	**Data base**	**NES** [a]	**Marker genes/related genes „Leading edge“** [b]
Lysosome	KEGG	1.94	LGMN
Complement and coagulation cascades	KEGG	1.86	C2, SERPING1, C1QA, C1QB
Lipid digestion mobilization and transport	Reactome	1.85	PLTP, APOE
Activation of chaperone genes by XBP1S	Reactome	1.76	
Arginine and proline metabolism	KEGG	1.69	
Iron uptake and transport	Reactome	1.68	HMOX1
Complement cascade	Reactome	1.66	C2, C1QA, C1QB
Extracellular matrix organization	Reactome	1.66	
MHC class II antigen presentation	Reactome	1.64	LGMN
Unfolded protein response	Reactome	1.63	
Diabetes pathways	Reactome	1.61	PLA2G7
Peptide ligand binding receptors	Reactome	1.60	CXCL9, C3AR1
Collagen formation	Reactome	1.60	
Response to elevated platelet cytosolic_CA2	Reactome	1.58	SERPING1
Toll like receptor signaling pathway	KEGG	1.55	CXCL9, CD14
Axon guidance	Reactome	1.55	NRP1, NRP2, GFRA2
Amino sugar and nucleotide sugar metabolism	KEGG	1.55	
Signal transduction by L1	Reactome	1.54	NRP1
Systemic lupus erythematosus	KEGG	1.53	C2, C1QA, C1QB
ECM receptor interaction	KEGG	1.53	SDC3

[a] NES was used for the arrangement of the top 20 signaling pathways with the strongest expression differences between ercDC_ccRCC&infMΦ_ascOvCa and control group because p-values and FDR values were very high. [b] ercDC_ccRCC marker genes and their related genes that belong to the so called “leading edge” genes of respective gene sets.

Table S13: Modules [40] and their assigned correlating/associated stimuli. Related to figure 7. We assigned a correlation/association of a stimulus with a module when the p-value was <0.05. If a correlation had a significance of p≤10^{-6}, solely this stimuli was listed as associated stimulus even if others had p-values <0.05. If none of the stimuli correlated with p≤10^{-6}, the two most significant stimuli (p<0.05) were used as module-associated stimuli. This approach is inspired by the connection of modules with M1- and M2-stimuli, respectively, described in Xue et al. The stimulus with the most significant correlation is listed first.

Module	Stimulus	
	positive correlation	negative correlation
1	LA, OA	IL-13, IL-4
2	n.d.	IL-13, IL-4
3	sLPS, M^b	IL-13, LiA
4	OA	IL-13, IL-4
5	PA	IL-4, IL-13
6	OA, LA	TPP, sLPS + IC
7	IFN-γ, PA	TPP, TNF+ PGE_2
8	IFN-γ	TPP*
9	IFN-γ + TNF, IFN-γ	TPP, TPP + IFN-β
10	IL-4, TNF	PA, LiA
11	IL-4	PA
12	IL-4, IFN-γ	TPP
13	IL-4, IL-13	TPP
14	IL-4	sLPS, upLPS + IC
15	IL-4	TPP*
16	PA	IL-4, TPP + IFN-γ
17	PA	IL-4, IFN-γ + TNF
18	PA	sLPS, TPP
19	PA	IL-4, upLPS
20	PA	TPP
21	PA	TPP + IFN-β, TPP
22	OA	TPP + IFN-β + IFN-γ, TPP + IFN-β
23	OA, LiA	TPP, sLPS
24	OA	TPP, TPP + IFN-β + IFN-γ
25	PA	sLPS, TPP
26	LiA	sLPS, TPP
27	LA, HDL	sLPS
28	OA, IL-4	sLPS
29	TNF+ PGE_2, TPP	M^b, IFN-γ
30	TPP	IL-4, M^b
31	PA	IL-4, M^b
32	TPP	IL-4, M^b
33	TPP	PA, OA
34	TPP, sLPS + IC	OA, LA
35	IFN-γ + TNF, sLPS + IFN-γ	OA, LA
36	TPP, P3C + PGE_2	OA, IFN-γ
37	IL-4, upLPS	PA
38	TPP, sLPS + IC	OA
39	TPP, PGE_2	PA
40	TPP, upLPS	PA
41	GC, PGE_2	PA, TNF
42	GC, M^b	PA, TNF + PGE_2
43	M^b, PGE_2	sLPS, IFN-γ + TNF
44	OA, GC	IFN-γ, TPP + IFN-β + IFN-γ
45	IL-4	PA, sLPS
46	IL-4	PA
47	IL-4, GC	PA
48	IL-4	PA, OA
49	n.d.	n.d.

*Only one stimulus possessed a p-value <0.05, but >10^{-6} for the module; n.d.: not detected; M^b: baseline macrophages

Supplemental experimental procedures

Characteristics of human myeloid cell types from different non-lymphoid tissues used for comparison with the ercDC transcriptome

Renal DCs and macrophages are thought to play a role in protecting the kidney tubulointerstitium from immune attack-induced injury through T-cell tolerizing mechanisms [67]. To gain insight if ercDCs might exhibit tolerizing features associated with tissue/tumor immune protection, we thought to compare the ercDC transcriptome to that of other myeloid cell types originating from tissues with a known tolerogenic milieu (table S2B). Of particular interest were the $CD11c^{HI}$ and $CD11c^{LO}$ decidual macrophages (previously designated decidual DCs) [31], found in the tolerogenic placenta during early pregnancy. Like ercDCs, they are decribed to co-express CD209, CD14 as well as CD163, and seemed to be capable of antigen presentation, $CD11c^{HI}$ macrophages mainly expressed genes associated with lipid metabolism and inflammation, while $CD11c^{LO}$ macrophages exhibited strong expression of genes associated with tissue modulation, which are important for embryo implantation, but also for tumor growth and progression. As an additional data set we selected transcriptomes of myeloid cells from gut and lung, considering that in these organs, the immune system is important to maintain the balance between immunity and peripheral tolerance. Three different DC subtypes are described in the lamina propria distinguished based on the expression of CD103 and Sirpα [68]). $CD103^{+}Sirp\alpha^{-}$ DCs are described to resemble $CD141^{+}$ DCs from blood and express marker associated with crosspresentation; $CD103^{+}Sirp\alpha^{+}$ DCs have a described similarity with $CD1c^{+}$ DCs from blood. They are known to induce T_H17 cells and express CD209 and genes associated with tolerizing functions. The third population, $CD103^{-}Sirp\alpha^{+}$ DCs, has strong similarity with monocyte-derived DCs (MoDCs) and is thought to effectively expand T_H1 cells. Concering myeloid cells from lung, two data sets were available [69,70]. They included macrophages isolated from non-smokers, smokers and COPD or asthma patients, respectively. Macrophages from smokers and COPD patients are described to express features associated with downregulating M1-associated proinflammatory genes and upregulating M2-associated regulatory genes compared to macrophages from non-smokers [69]. They expressed high amounts of MMPs, a characteristic also described for macrophages in the tumors.

Further, we selected data sets of human tumor samples. These included, macrophages from gastrointestinal stromal tumors (GIST) [71] and inflammatory macrophages and DCs from ascites of ovarian cancer patients [72]. Notably, the macrophages from GIST are described to have antitumoral, proinflammatory features resembling M1-macrophages. As representative for DCs with the capability of crosspresention and activation of $CD8^{+}$ T cells [75,76] we selected the data sets of $CD141^{+}$ DCs from Lindstedt et al. [74].

Supplemental references (numbers according to the listing in the main text)

10. Mosser, D.M.; Edwards, J.P. Exploring the full spectrum of macrophage activation. Nat Rev Immunol 2008, 8, 958-969, doi:10.1038/nri2448.

31. Houser, B.L.; Tilburgs, T.; Hill, J.; Nicotra, M.L.; Strominger, J.L. Two unique human decidual macrophage populations. J Immunol 2011, 186, 2633-2642, doi:10.4049/jimmunol.1003153.

32. Martinez, F.O.; Gordon, S.; Locati, M.; Mantovani, A. Transcriptional profiling of the human monocyte-to-macrophage differentiation and polarization: new molecules and patterns of gene expression. J Immunol 2006, 177, 7303-7311, doi:10.4049/jimmunol.177.10.7303.

34. Tibshirani, R.; Hastie, T.; Narasimhan, B.; Chu, G. Diagnosis of multiple cancer types by shrunken centroids of gene expression. Proc Natl Acad Sci U S A 2002, 99, 6567-6572, doi:10.1073/pnas.082099299.

36. Breuer, K.; Foroushani, A.K.; Laird, M.R.; Chen, C.; Sribnaia, A.; Lo, R.; Winsor, G.L.; Hancock, R.E.; Brinkman, F.S.; Lynn, D.J. InnateDB: systems biology of innate immunity and beyond--recent updates and continuing curation. Nucleic Acids Res 2013, 41, D1228-1233, doi:10.1093/nar/gks1147.

37. Lynn, D.J.; Chan, C.; Naseer, M.; Yau, M.; Lo, R.; Sribnaia, A.; Ring, G.; Que, J.; Wee, K.; Winsor, G.L.; et al. Curating the innate immunity interactome. BMC Syst Biol 2010, 4, 117, doi:10.1186/1752-0509-4-117.

38. Lynn, D.J.; Winsor, G.L.; Chan, C.; Richard, N.; Laird, M.R.; Barsky, A.; Gardy, J.L.; Roche, F.M.; Chan, T.H.; Shah, N.; et al. InnateDB: facilitating systems-level analyses of the mammalian innate immune response. Mol Syst Biol 2008, 4, 218, doi:10.1038/msb.2008.55.

39. Subramanian, A.; Tamayo, P.; Mootha, V.K.; Mukherjee, S.; Ebert, B.L.; Gillette, M.A.; Paulovich, A.; Pomeroy, S.L.; Golub, T.R.; Lander, E.S.; et al. Gene set enrichment analysis: a knowledge-based approach for interpreting genome-wide expression profiles. Proc Natl Acad Sci U S A 2005, 102, 15545-15550, doi:10.1073/pnas.0506580102.

40. Xue, J.; Schmidt, S.V.; Sander, J.; Draffehn, A.; Krebs, W.; Quester, I.; De Nardo, D.; Gohel, T.D.; Emde, M.; Schmidleithner, L.; et al. Transcriptome-based network analysis reveals a spectrum model of human macrophage activation. Immunity 2014, 40, 274-288, doi:10.1016/j.immuni.2014.01.006.

44. Maruschke, M.; Hakenberg, O.W.; Koczan, D.; Zimmermann, W.; Stief, C.G.; Buchner, A. Expression profiling of metastatic renal cell carcinoma using gene set enrichment analysis. Int J Urol 2014, 21, 46-51, doi:10.1111/iju.12183.

45. Maruschke, M.; Reuter, D.; Koczan, D.; Hakenberg, O.W.; Thiesen, H.J. Gene expression analysis in clear cell renal cell carcinoma using gene set enrichment analysis for biostatistical management. BJU Int. 2011, 108, E29-35, doi:10.1111/j.1464-410X.2010.09794.x.

56. Gustafsson, C.; Mjosberg, J.; Matussek, A.; Geffers, R.; Matthiesen, L.; Berg, G.; Sharma, S.; Buer, J.; Ernerudh, J. Gene expression profiling of human decidual macrophages: evidence for immunosuppressive phenotype. PLoS One 2008, 3, e2078, doi:10.1371/journal.pone.0002078.

57. Wang, T.; Ge, Y.; Xiao, M.; Lopez-Coral, A.; Azuma, R.; Somasundaram, R.; Zhang, G.; Wei, Z.; Xu, X.; Rauscher, F.J., 3rd; et al. Melanoma-derived conditioned media efficiently induce the differentiation of monocytes to macrophages that display a highly invasive gene signature. Pigment Cell Melanoma Res 2012, 25, 493-505, doi:10.1111/j.1755-148X.2012.01005.x.

67. Kurts, C.; Heymann, F.; Lukacs-Kornek, V.; Boor, P.; Floege, J. Role of T cells and dendritic cells in glomerular immunopathology. Semin Immunopathol 2007, 29, 317-335, doi:10.1007/s00281-007-0096-x.

68. Watchmaker, P.B.; Lahl, K.; Lee, M.; Baumjohann, D.; Morton, J.; Kim, S.J.; Zeng, R.; Dent, A.; Ansel, K.M.; Diamond, B.; et al. Comparative transcriptional and functional profiling defines conserved programs of intestinal DC differentiation in humans and mice. Nat Immunol 2014, 15, 98-108, doi:10.1038/ni.2768.

69. Shaykhiev, R.; Krause, A.; Salit, J.; Strulovici-Barel, Y.; Harvey, B.G.; O'Connor, T.P.; Crystal, R.G. Smoking-dependent reprogramming of alveolar macrophage polarization: implication for pathogenesis of chronic obstructive pulmonary disease. J Immunol 2009, 183, 2867-2883, doi:10.4049/jimmunol.0900473.

70. Woodruff, P.G.; Koth, L.L.; Yang, Y.H.; Rodriguez, M.W.; Favoreto, S.; Dolganov, G.M.; Paquet, A.C.; Erle, D.J. A distinctive alveolar macrophage activation state induced by cigarette smoking. Am J Respir Crit Care Med 2005, 172, 1383-1392, doi:10.1164/rccm.200505-686OC.

71. Cavnar, M.J.; Zeng, S.; Kim, T.S.; Sorenson, E.C.; Ocuin, L.M.; Balachandran, V.P.; Seifert, A.M.; Greer, J.B.; Popow, R.; Crawley, M.H.; et al. KIT oncogene inhibition drives intratumoral macrophage M2 polarization. J Exp Med 2013, 210, 2873-2886, doi:10.1084/jem.20130875.

72. Segura, E.; Amigorena, S. Inflammatory dendritic cells in mice and humans. Trends Immunol 2013, 34, 440-445, doi:10.1016/j.it.2013.06.001.

73. Segura, E.; Touzot, M.; Bohineust, A.; Cappuccio, A.; Chiocchia, G.; Hosmalin, A.; Dalod, M.; Soumelis, V.; Amigorena, S. Human inflammatory dendritic cells induce Th17 cell differentiation. Immunity 2013, 38, 336-348, doi:10.1016/j.immuni.2012.10.018.

74. Lindstedt, M.; Lundberg, K.; Borrebaeck, C.A. Gene family clustering identifies functionally associated subsets of human in vivo blood and tonsillar dendritic cells. J Immunol 2005, 175, 4839-4846, doi:10.4049/jimmunol.175.8.4839.

75. Bachem, A.; Guttler, S.; Hartung, E.; Ebstein, F.; Schaefer, M.; Tannert, A.; Salama, A.; Movassaghi, K.; Opitz, C.; Mages, H.W.; et al. Superior antigen cross-presentation and XCR1 expression define human CD11c+CD141+ cells as homologues of mouse CD8+ dendritic cells. J Exp Med 2010, 207, 1273-1281, doi:10.1084/jem.20100348.

76. Mittag, D.; Proietto, A.I.; Loudovaris, T.; Mannering, S.I.; Vremec, D.; Shortman, K.; Wu, L.; Harrison, L.C. Human dendritic cell subsets from spleen and blood are similar in phenotype and function but modified by donor health status. J Immunol 2011, 186, 6207-6217, doi:10.4049/jimmunol.1002632.

85. Chittezhath, M.; Dhillon, M.K.; Lim, J.Y.; Laoui, D.; Shalova, I.N.; Teo, Y.L.; Chen, J.; Kamaraj, R.; Raman, L.; Lum, J.; et al. Molecular profiling reveals a tumor-promoting phenotype of monocytes and macrophages in human cancer progression. Immunity 2014, 41, 815-829, doi:10.1016/j.immuni.2014.09.014.
