# Supplementary material for: Dendritic Cells or Macrophages? The Microenvironment of Human Clear Cell Renal Cell Carcinoma Imprints a Mosaic Myeloid Subtype Associated with Patient Survival
Source: Cells. 2022 Oct 19;11(20):3289. doi: 10.3390/cells11203289 (PMC9600747; doi:10.3390/cells11203289)
Supplement: Supplementary file 1 [file cells-11-03289-s001.zip › ercDC_cells submission_Table S10.pdf]

Table S10: Upregulated ercDC_ccRCC DEGs. Listed are genes significantly (adjusted p<0.05) upregulated between ercDC_ccRCC&infMΦ_ascOvCa and control group (431 of 788 ercDC_ccRCC DEGs). Genes are sorted acc. to increasing adjusted p-values. Genes that also belong to ercDC_ccRCC marker genes are in bold green. Genes related to ercDC_ccRCC marker genes are green. Genes mentioned in main text are in grey.

Gene symbol	Entrez ID	norm. expr.values (log2) ercDC_ccRCC&infMΦ_ascOvCa	norm. expr.values (log2) control group	logFC	adjusted p-value
**APOC1**	341	11.82	10.04	1.78	0.00003
**FOLR2**	2350	8.7	7.31	1.39	0.00003
**GAL3ST4**	79690	8.01	6.59	1.42	0.00006
**SDC3**	9672	7.65	6.42	1.23	0.00006
**ME1**	4199	9.59	8.13	1.46	0.00008
**C2**	717	8.52	7.07	1.46	0.00008
**CCL8**	6355	8.53	6.57	1.96	0.00012
**CD209**	30835	8.44	7.05	1.39	0.00012
**PLTP**	5360	9.71	7.71	2	0.00019
SLC2A5	6518	6.85	5.53	1.32	0.00019
ITGA9	3680	6.19	5.28	0.9	0.00019
**SLCO2B1**	11309	9.65	7.79	1.86	0.00027
**GPNMB**	10457	11.22	9.09	2.13	0.00031
**ABCC5**	10057	7.94	7.1	0.84	0.00031
AP2A2	161	8.97	8.28	0.69	0.00031
TNS3	64759	9.41	8.79	0.63	0.00031
**SEPP1**	6414	9.11	6.69	2.42	0.00034
**NRP2**	8828	8.12	6.62	1.5	0.00034
CXCL12	6387	6.52	5.73	0.79	0.00034
LHFPL2	10184	10.97	9.58	1.38	0.00036
**SLC38A6**	145389	10.17	9.02	1.15	0.00036
CMKLR1	1240	8.61	7.16	1.46	0.0004
OLFML2B	25903	7.65	6.34	1.31	0.0004
**NRP1**	8829	9.98	8.25	1.73	0.00042
**SLC7A8**	23428	7.9	6.27	1.63	0.00042
SLC38A7	55238	7.49	6.79	0.7	0.00042
LILRB5	10990	6.87	5.9	0.97	0.00044
**NUPR1**	26471	9.85	8.51	1.34	0.00047
ACP2	53	9.95	9.05	0.91	0.00047
EPHB2	2048	6.71	5.8	0.91	0.0005
WBP5	51186	8.96	7.98	0.98	0.00051
CTSL	1514	12.28	10.97	1.3	0.00052
SLC36A1	206358	8.1	7.41	0.69	0.00057
**FABP3**	2170	8.4	6.86	1.54	0.00059
**DAB2**	1601	9.9	8.39	1.51	0.00059
MMP14	4323	8.05	6.92	1.14	0.00059
KAL1	3730	8.95	7.78	1.16	0.00065
**APOE**	348	11.05	9.23	1.82	0.00068
ABCA1	19	9.63	8.46	1.17	0.00068
FRMD4A	55691	7.92	6.81	1.11	0.00068
TEC	7006	6.45	5.96	0.49	0.00068
**SIGLEC1**	6614	9.68	8.23	1.45	0.0007
**CCL18**	6362	11.42	9.62	1.8	0.00076
**ADAMDEC1**	27299	9.06	7.33	1.73	0.00077
**C1QA**	712	11.83	10.41	1.42	0.00081
DOCK4	9732	9.29	8.04	1.25	0.00081
**CRYAB**	1410	6.75	5.68	1.08	0.00083
NR1H3	10062	9.01	7.95	1.06	0.00083
RAB3IL1	5866	7.3	6.76	0.54	0.00093

TREM2	54209	9.99	8.39	1.6	0.00099
PLOD2	5352	5.22	4.27	0.95	0.00099
SLC1A3	6507	8.06	6.67	1.39	0.00109
**RHOBTB3**	22836	7.21	6.07	1.14	0.00109
DRAM1	55332	10.26	9.37	0.89	0.00109
HS3ST1	9957	6.9	5.74	1.16	0.00109
**MAF**	4094	8.17	6.88	1.28	0.00111
**C1QB**	713	12.33	10.92	1.42	0.00112
CD64	2209	6.96	5.79	1.18	0.00112
**IL2RA**	3559	6.85	5.69	1.16	0.00112
**ARHGAP12**	94134	7.61	6.79	0.82	0.00112
PSD3	23362	7.92	6.81	1.11	0.00115
TNFRSF11A	8792	6.77	5.81	0.96	0.00119
CTSB	1508	12.23	11.32	0.91	0.00119
ADAP2	55803	9.85	9.02	0.83	0.00119
CFH	3075	5.72	5.09	0.63	0.00127
RNASE1	6035	10.53	8.67	1.86	0.00135
ITGAV	3685	10.5	9.47	1.03	0.00135
**SCAMP5**	192683	6.18	5.54	0.64	0.00149
TCN2	6948	8.26	7.45	0.81	0.00149
TDRKH	11022	6.33	5.65	0.68	0.00149
**SERPING1**	710	11.43	9.99	1.45	0.0015
IL10	3586	6.98	5.89	1.1	0.00165
MMP19	4327	9.5	8.23	1.27	0.00165
**MS4A4A**	51338	10.52	9.3	1.21	0.00193
TMEM51	55092	8.87	7.95	0.92	0.00193
MYO7A	4647	5.97	5.27	0.71	0.00193
ZFYVE26	23503	9.17	8.55	0.62	0.00193
ITSN1	6453	7.87	6.9	0.97	0.00199
SCARB2	950	9.58	8.67	0.91	0.00199
CFB	629	8.06	6.82	1.25	0.00206
EDNRB	1910	5.53	4.79	0.74	0.00207
CD163	9332	8.32	6.79	1.53	0.00207
PDCD1LG2	80380	7.64	6.48	1.16	0.00207
STAB1	23166	8.69	7.56	1.13	0.00207
ADAM9	8754	10.03	9.1	0.93	0.00207
SNX24	28966	7.07	6.31	0.76	0.00207
CD81	975	12.34	11.6	0.75	0.00207
CD28	940	4.91	4.28	0.63	0.00207
FLCN	201163	6.85	6.34	0.51	0.00207
C3	718	9.97	8.43	1.54	0.00223
MITF	4286	8.4	7.7	0.7	0.00228
CRYBB1	1414	6.2	5.7	0.5	0.00228
EPAS1	2034	9.11	7.93	1.18	0.00244
GLUL	2752	10.8	9.74	1.06	0.00246
MERTK	10461	7.93	6.5	1.43	0.0025
ADORA3	140	7.24	6.02	1.22	0.0025
TRPV4	59341	6.56	5.98	0.57	0.00257
PTPRM	5797	8.37	7.57	0.79	0.0026
BMP2K	55589	8.97	8.16	0.81	0.00262
APPL2	55198	7.39	6.79	0.6	0.00263
KIAA0226L	80183	8.69	7.6	1.09	0.00267
NPC1	4864	9.11	8.04	1.07	0.00267
LAIR1	3903	9.89	8.86	1.02	0.00267
VAT1	10493	10.31	9.55	0.76	0.00267
LGALS3BP	3959	10.19	9.19	1	0.0028

GNPDA1	10007	9.75	9.19	0.56	0.00285
IDH1	3417	10.69	9.84	0.85	0.00288
HNMT	3176	9.35	8.36	1	0.00288
**LGMN**	5641	11.67	10.11	1.56	0.00288
CD84	8832	9.11	7.84	1.27	0.0029
SGMS1	259230	8.66	7.91	0.75	0.0033
CTSD	1509	11.32	10.42	0.9	0.00346
BCL2L1	598	7.62	6.72	0.9	0.00348
STX4	6810	9.78	9.24	0.53	0.00355
SDS	10993	7.87	6.8	1.07	0.00369
BNIP3	664	8.06	7.01	1.05	0.00369
SPP1	6696	11.15	8.64	2.51	0.00375
MPP1	4354	10.58	9.99	0.59	0.00385
	3569	7.68	6.29	1.39	0.00389
NPL	80896	9	7.94	1.06	0.00389
STARD13	90627	6.22	5.7	0.52	0.00395
FCGR1B	2210	11.09	9.8	1.29	0.004
MARCKS	4082	10.25	8.94	1.31	0.00412
TCF12	6938	9.19	8.63	0.56	0.00412
PLA2G15	23659	8.76	8.07	0.68	0.00421
CCL4	6351	11.32	9.56	1.76	0.00432
VSIG4	11326	11.86	10.59	1.27	0.00433
CCL2	6347	10.46	8.71	1.76	0.00459
MGAT4A	11320	9.3	8.1	1.2	0.00459
RND3	390	8.82	7.69	1.13	0.00459
RGL1	23179	9.28	8.17	1.11	0.00459
LIPA	3988	12.69	12	0.69	0.00459
COLGALT1	79709	8.91	8.32	0.59	0.00459
CREG1	8804	12.04	11.5	0.53	0.00459
LRP1	4035	8.64	7.99	0.65	0.00463
CYFIP1	23191	11.25	10.73	0.52	0.00463
CPM	1368	9.7	8.68	1.02	0.00465
CALU	813	8.49	7.88	0.61	0.00475
MYO5A	4644	8.96	8.43	0.53	0.0049
**IGF1**	3479	7.08	5.85	1.23	0.00491
MAPK13	5603	8.2	7.49	0.72	0.00491
GALC	2581	9.19	8.47	0.72	0.00491
FZD5	7855	7.25	6.59	0.67	0.00504
SLC6A8	6535	6.72	6.19	0.53	0.00504
DYNLT3	6990	10.21	9.72	0.49	0.0054
PLIN2	123	10.84	9.93	0.92	0.00547
METTL1	4234	7.44	6.86	0.58	0.00561
EYA2	2139	5.29	4.87	0.42	0.00574
CXCL10	3627	10.24	8.15	2.08	0.00575
CXCL2	2920	10.79	9.38	1.41	0.00582
TFRC	7037	10.35	9.33	1.03	0.00582
PLXNA3	55558	6.46	5.86	0.6	0.00582
ATP13A2	23400	7.37	6.72	0.65	0.00605
LYVE1	10894	6.39	5.4	1	0.0066
OLR1	4973	11	9.4	1.6	0.00663
RBM47	54502	9.35	8.69	0.65	0.00668
FN1	2335	10.06	8.12	1.93	0.00675
MARCO	8685	10.44	9.42	1.03	0.00683
FAM13A	10144	8.11	7.33	0.78	0.00683
AP1B1	162	8.95	8.46	0.49	0.00683
CAMSAP2	23271	7.83	7.03	0.8	0.00687

IGFBP4	3487	7.04	6.45	0.59	0.00698
P4HA2	8974	7.5	6.97	0.53	0.00711
ATP6V1C1	528	8.56	7.86	0.7	0.00723
ENOSF1	55556	8.16	7.49	0.67	0.00754
GPR65	8477	10.49	9.78	0.71	0.0076
OLFML3	56944	6.1	5.5	0.6	0.0076
SLC11A2	4891	8.37	7.79	0.57	0.0076
PLAT	5327	5.3	4.77	0.52	0.0076
PLXND1	23129	9.37	8.85	0.52	0.0076
CTSZ	1522	11.03	10.49	0.54	0.00797
CXCL1	2919	8.55	7.33	1.22	0.00807
ETV5	2119	7.67	6.56	1.11	0.00812
DNASE2	1777	8.54	7.76	0.79	0.00812
CD204	4481	9.74	8.14	1.6	0.00817
IFI27	3429	9.65	8.19	1.47	0.00818
ASPH	444	8.16	7.41	0.75	0.00818
PLAU	5328	8.77	7.62	1.15	0.00827
HOMER3	9454	7.32	6.71	0.61	0.00856
ITPR2	3709	9.18	8.33	0.85	0.00876
FABP5	2171	11.79	10.75	1.03	0.00877
FNDC3B	64778	8.84	8.13	0.71	0.00969
GPR137B	7107	10.84	10.08	0.76	0.00992
ANGPTL4	51129	6.29	5.73	0.56	0.00992
TIMP2	7077	10.99	10.48	0.51	0.00992
GLA	2717	10.87	10.29	0.58	0.0101
PI4K2A	55361	8.75	8.17	0.58	0.01016
KCNMA1	3778	8.67	7.51	1.15	0.01024
ELL2	22936	8.79	7.84	0.96	0.01035
MMP2	4313	7.67	6.67	0.99	0.01036
DYRK4	8798	7.75	7.32	0.43	0.01036
CP	1356	5.03	4.2	0.83	0.01039
A2M	2	11.18	9.48	1.7	0.0104
FRMD4B	23150	8.6	7.67	0.93	0.0104
PLEKHO2	80301	9.95	9.46	0.49	0.01079
HAMP	57817	8.1	6.82	1.29	0.01099
ADAMTS2	9509	5.74	5.3	0.44	0.01115
SCIN	85477	5.25	4.52	0.73	0.01139
IQCG	84223	7.12	6.69	0.43	0.01139
LINC00597	81698	5.16	4.73	0.43	0.01139
LILRB4	11006	9.02	8.24	0.77	0.01143
PEAK1	79834	8.04	7.55	0.49	0.0118
SLC16A10	117247	7.59	6.48	1.11	0.01183
CTSA	5476	11.29	10.74	0.55	0.01183
ACE	1636	6.14	5.66	0.48	0.01183
ATG7	10533	7.31	6.79	0.52	0.01194
MKNK1	8569	9.51	8.86	0.65	0.012
PLD3	23646	10.31	9.49	0.82	0.01204
CXCL11	6373	7.07	5.48	1.59	0.01226
SGPL1	8879	8.52	7.98	0.54	0.0123
FPR3	2359	10.57	9.15	1.42	0.01241
RCN3	57333	6.72	6.32	0.41	0.01252
PMP22	5376	10.3	9.2	1.1	0.0127
PROS1	5627	8.22	7.57	0.65	0.01279
RAB13	5872	10.31	9.57	0.74	0.01288
CDR1	1038	4.36	3.84	0.51	0.01288
ECM1	1893	7.47	6.77	0.71	0.01295

WASF1	8936	4.81	4.31	0.5	0.01313
ATP2A2	488	8.51	8.07	0.44	0.01316
CADM1	23705	7.64	6.36	1.28	0.0132
FARP1	10160	7.68	7.15	0.53	0.0132
WDFY3	23001	7.36	6.77	0.59	0.01333
SLC37A4	2542	7.25	6.86	0.38	0.01348
RNASE2	6036	7.92	6.95	0.97	0.01351
ALG9	79796	7.64	7.2	0.44	0.01352
HSPB1	3315	10.96	10.13	0.83	0.01353
HSP90B1	7184	9.34	8.67	0.67	0.01356
ABCG1	9619	8.03	7.24	0.79	0.01363
DENND2D	79961	9	8.54	0.46	0.01373
PDIA5	10954	7.16	6.67	0.49	0.01383
MMP9	4318	9.81	8.47	1.34	0.01402
SAMD4A	23034	7.76	7.11	0.65	0.01404
PPARG	5468	9.26	8.4	0.86	0.01425
TNS1	7145	8.89	7.65	1.23	0.01452
SCCPDH	51097	8.5	7.94	0.56	0.01541
CH25H	9023	7.89	6.63	1.26	0.01549
ADM	133	9.08	8.12	0.96	0.01552
ST8SIA4	7903	7.17	6.48	0.69	0.01595
SCD	6319	9.38	8.19	1.19	0.01618
ACP5	54	11.01	10.19	0.82	0.01647
PCOLCE2	26577	8.39	7.58	0.81	0.01656
9月-08	23176	6.95	6.52	0.43	0.01656
PLEKHM2	23207	8.46	8.04	0.42	0.01656
**CXCL9**	4283	9.73	7.88	1.85	0.01704
IL15RA	3601	8.23	7.64	0.6	0.01704
APBB3	10307	7.35	6.91	0.45	0.01737
ZFP36L1	677	9.84	9.12	0.72	0.01763
AMPD3	272	8.78	8.03	0.74	0.01765
SLC4A7	9497	7.41	6.78	0.63	0.01765
TBC1D2	55357	8.5	7.89	0.6	0.01765
SLC2A8	29988	6.73	6.27	0.46	0.01765
EOGT	285203	7.95	7.36	0.59	0.01779
NFE2L1	4779	9.28	8.84	0.44	0.01779
LPAR6	10161	10.06	9.23	0.83	0.0179
RENBP	5973	7.58	7.07	0.51	0.0179
FUCA1	2517	11.39	10.57	0.82	0.01799
MAFB	9935	11.67	10.74	0.93	0.01852
ZDHHC14	79683	7.2	6.72	0.48	0.01852
BAMBI	25805	6	5.5	0.5	0.01859
ABCC3	8714	8.43	7.69	0.73	0.01883
TPX2	22974	6.15	5.56	0.59	0.01883
GBAP1	2630	8.1	7.74	0.37	0.01883
GNPTAB	79158	8.78	8.29	0.49	0.01894
CXCL3	2921	9.27	8.22	1.05	0.01896
CTSC	1075	10.9	10.12	0.78	0.0196
CD82	3732	8.49	7.84	0.65	0.0198
RACGAP1	29127	7.72	7.16	0.55	0.01998
P2RX4	5025	9.7	9.03	0.67	0.02036
TGFBI	7045	12.22	11.66	0.56	0.02045
LXN	56925	7.38	6.86	0.52	0.02045
PPAP2B	8613	9	8	1	0.02048
SPHK1	8877	7.41	6.77	0.65	0.02048
P4HB	5034	9.86	9.14	0.72	0.02081

IER3	8870	11.54	10.32	1.21	0.02128
PLOD1	5351	8.6	8.22	0.38	0.02128
ITGB5	3693	6.83	6.05	0.77	0.02155
TMEM140	55281	8.75	8.22	0.53	0.02162
ATP6V0A1	535	8.86	8.32	0.54	0.02167
BNC2	54796	5.75	5.25	0.49	0.02167
NENF	29937	8.57	8.18	0.39	0.02167
RRAGD	58528	9.86	9.27	0.6	0.0217
CD59	966	8.6	7.77	0.83	0.02187
ACVRL1	94	7.62	7.1	0.52	0.02206
GAA	2548	9.63	9.14	0.48	0.02257
CD80	941	8.19	7.1	1.09	0.02272
CTNS	1497	8.43	7.83	0.6	0.02272
SLC31A1	1317	9.1	8.56	0.54	0.02272
NABP1	64859	9.35	8.72	0.63	0.02296
PDIA4	9601	9.21	8.68	0.54	0.02296
HSPA5	3309	10.33	9.79	0.54	0.02311
COLEC12	81035	8.99	8.14	0.85	0.02311
MDFIC	29969	8.23	7.58	0.65	0.02311
BLNK	29760	9.14	8.1	1.04	0.02312
MMP7	4316	6.57	5.61	0.96	0.02329
AAK1	22848	8.95	8.53	0.42	0.0235
CCL7	6354	6.43	5.57	0.86	0.02353
ARHGAP6	395	7.24	6.78	0.46	0.02353
FCGRT	2217	10.71	10.24	0.48	0.02378
EPHX1	2052	8.02	7.43	0.58	0.02387
B2M	567	9.52	9.13	0.39	0.02387
TRIP6	7205	8.46	8.06	0.4	0.02393
CD14	929	11.49	10.52	0.97	0.02409
ATXN1	6310	8.59	7.92	0.67	0.02411
HTRA1	5654	7.6	6.95	0.65	0.02411
LAMP1	3916	9.72	8.81	0.91	0.02412
GGCX	2677	7.5	7.12	0.37	0.02418
GATM	2628	8.5	7.42	1.08	0.02487
PXDC1	221749	7.73	7.07	0.66	0.0252
SERPINE1	5054	7.06	6.24	0.83	0.02551
IKBKE	9641	7.31	6.93	0.38	0.02551
DGKH	160851	5.47	5.1	0.37	0.02551
PAX8	7849	6.53	5.95	0.58	0.02585
PRUNE2	158471	6.94	5.97	0.96	0.02606
ENG	2022	9.2	8.5	0.7	0.02606
C1R	715	6.65	6.08	0.57	0.02606
CD2AP	23607	7.79	7.24	0.56	0.02606
TUBG1	7283	8.36	7.89	0.48	0.02606
KIAA1279	26128	7.78	7.38	0.4	0.02606
ERI2	112479	7.34	6.93	0.42	0.02618
AHI1	54806	7.36	6.7	0.65	0.02626
MSRB2	22921	8.5	8.06	0.44	0.0265
DHRS3	9249	9.01	8.16	0.85	0.02654
SERPINH1	871	7.86	7.23	0.63	0.02736
MR1	3140	7.46	6.96	0.5	0.02736
ME2	4200	9.09	8.6	0.5	0.02736
DSC2	1824	8.48	7.6	0.88	0.02772
LAMP2	3920	9.89	9.34	0.55	0.02806
MORF4L2	9643	6.95	6.43	0.52	0.02842
CA12	771	6.19	5.47	0.72	0.02854

HSD17B14	51171	7.13	6.61	0.51	0.02854
QKI	9444	8.66	8.14	0.52	0.02854
RARRES1	5918	7.02	6.12	0.9	0.02866
CALR	811	10.03	9.35	0.69	0.02887
LUM	4060	4.74	4.2	0.54	0.02912
C10orf10	11067	5.87	5.43	0.45	0.02912
KIAA1199	57214	5.46	5.08	0.37	0.02912
COL1A1	1277	7.07	6.52	0.55	0.02928
PVRL2	5819	7.52	6.88	0.64	0.02929
SMURF2	64750	7.89	7.28	0.61	0.02943
CCL13	6357	7.34	6.43	0.91	0.02989
NTAN1	123803	9.16	8.68	0.49	0.02989
TFEC	22797	9.53	8.68	0.85	0.03004
GCNT1	2650	7.69	7.15	0.54	0.03059
PLD1	5337	6.63	5.95	0.67	0.03059
CD9	928	9.62	8.55	1.07	0.03123
EPB41L2	2037	9.04	8.38	0.66	0.03123
EIF4A3	9775	10.54	10.09	0.44	0.03123
CCRL2	9034	8.92	8.17	0.75	0.03178
ARHGAP10	79658	7.96	7.5	0.46	0.03203
PDGFRL	5157	4.99	4.59	0.4	0.033
EPB41L3	23136	10.43	9.8	0.64	0.03305
CD24	100133941	6.34	5.38	0.96	0.0332
FAM168A	23201	7.61	7.21	0.4	0.03354
KLF6	1316	9.57	8.89	0.67	0.03429
DSE	29940	11.12	10.48	0.65	0.03434
MELK	9833	6.65	6.07	0.58	0.03434
CD68	968	10.56	10.16	0.4	0.03436
MDC1	9656	7.22	6.82	0.4	0.03443
CLN6	54982	7.98	7.48	0.5	0.03482
COL3A1	1281	5.74	5.32	0.42	0.03482
IBSP	3381	5.37	4.95	0.41	0.03482
RYR1	6261	7.13	6.53	0.6	0.03497
TRPM2	7226	6.98	6.55	0.43	0.03592
ASIP	434	5.23	4.84	0.39	0.03598
PDIA3	2923	10.24	9.89	0.35	0.03652
BHLHE41	79365	8.47	7.43	1.03	0.03661
CEP55	55165	6.05	5.34	0.72	0.03673
HIVEP3	59269	7.13	6.5	0.62	0.03716
PRDX4	10549	9.73	9.32	0.4	0.03728
STAT1	6772	10.14	9.5	0.64	0.03733
APOL1	8542	7.99	7.39	0.6	0.03738
ARHGEF11	9826	7.45	6.96	0.49	0.03756
TGM2	7052	9.77	8.9	0.87	0.03801
MT1E	4493	9.64	8.97	0.67	0.03801
ARMCX1	51309	8.18	7.69	0.49	0.03801
LAMC1	3915	6.79	6.16	0.63	0.03811
CYB5A	1528	9.61	9.19	0.42	0.03819
MGAT5	4249	9.45	9.06	0.39	0.03838
CDCP1	64866	8.31	7.59	0.72	0.03845
DENND4C	55667	9.34	8.86	0.48	0.03845
TSPAN4	7106	8.42	7.8	0.62	0.0385
TLR7	51284	9.49	8.38	1.11	0.03866
MGLL	11343	9.67	9.1	0.57	0.03893
GNG12	55970	6.91	6.35	0.56	0.03893
RPS27L	51065	8.05	7.53	0.52	0.03905

RASGRP3	25780	7.95	7.05	0.9	0.03919
TNFAIP3	7128	10.77	9.97	0.8	0.03919
RBP4	5950	7.85	7.27	0.58	0.03919
COL1A2	1278	6.13	5.7	0.43	0.03919
SASH1	23328	9.01	8.35	0.66	0.03928
THBS3	7059	6.09	5.78	0.31	0.03936
PLOD3	8985	9.36	9.03	0.33	0.03973
SGK1	6446	12.04	11.46	0.58	0.04045
SLC39A8	64116	8.06	7.01	1.05	0.04047
HMOX1	3162	11.21	10.54	0.68	0.04047
LIMK2	3985	7.4	6.87	0.53	0.04047
LY96	23643	10.77	10.36	0.4	0.04047
KIFC3	3801	6.08	5.71	0.36	0.04115
CD151	977	8.58	8.06	0.52	0.04142
RRBP1	6238	7.88	7.48	0.41	0.04143
MILR1	284021	8.3	7.76	0.54	0.04155
FER	2241	6.19	5.8	0.39	0.04155
C3AR1	719	11.49	10.56	0.93	0.04161
B3GALNT1	8706	6.44	5.77	0.67	0.04202
LAP3	51056	11.47	11.01	0.46	0.04242
SPATA7	55812	6.01	5.62	0.39	0.04244
FAM114A1	92689	6.73	6.28	0.46	0.0434
ATRN	8455	7.54	7.18	0.36	0.0437
CCL20	6364	9.16	7.78	1.38	0.04372
MT2A	4502	12.07	11.5	0.58	0.04399
MUC1	4582	6.51	6.1	0.41	0.04402
GIMAP6	474344	8.04	7.24	0.8	0.04425
CCND1	595	6.73	6.03	0.7	0.04425
UAP1L1	91373	6.93	6.55	0.38	0.04426
GADD45B	4616	9.53	8.87	0.66	0.04447
CBR1	873	8.19	7.83	0.36	0.04447
NUCB1	4924	8.87	8.33	0.55	0.04452
MT1G	4495	10.37	9.63	0.75	0.04487
NLK	51701	7.5	7.11	0.39	0.04579
IGFBP3	3486	6.52	5.93	0.59	0.04714
GADD45G	10912	6.77	6.27	0.5	0.04714
VCAM1	7412	6.21	5.5	0.71	0.04716
PRDM1	639	8.73	7.91	0.83	0.04729
KCNJ5	3762	6.19	5.77	0.42	0.04753
ABL2	27	7.44	6.84	0.61	0.04865
STEAP3	55240	7.25	6.77	0.48	0.04884
CDH6	1004	5.62	5.24	0.38	0.04884
SREBF1	6720	7.44	7.11	0.33	0.04892
APOL2	23780	7.4	7.04	0.36	0.04933
FGFR1	2260	6.54	6.12	0.43	0.04939
TMEM180	79847	7.48	7.12	0.35	0.0495
