# Supplementary material for: Dendritic Cells or Macrophages? The Microenvironment of Human Clear Cell Renal Cell Carcinoma Imprints a Mosaic Myeloid Subtype Associated with Patient Survival
Source: Cells. 2022 Oct 19;11(20):3289. doi: 10.3390/cells11203289 (PMC9600747; doi:10.3390/cells11203289)
Supplement: Supplementary file 1 [file cells-11-03289-s001.zip › ercDC_cells submission_Table S11.pdf]

Table S11: Downregulated ercDC_ccRCC DEGs. Listed are genes significantly (adjusted p<0.05) downregulated between ercDC_ccRCC&infMΦ_ascOvCa and control group (357 of 788 ercDC_ccRCC DEGs). Genes are sorted acc. to increasing adjusted p-values. Genes that belong to ercDC_ccRCC marker genes are in bold green. Genes related to ercDC_ccRCC marker genes are in green. Genes mentioned in main text are in grey

**Gene symbol**	**Entrez ID**	**norm. expr. values (log) ercDC_RCC&infMΦ_ascOvCa**	**norm. expr.values (log) control group**	**logFC**	**adjusted p-value**
**FAM65B**	9750	5.16	6.4	-1.23	0.00034
RPS9	6203	11.06	11.73	-0.67	0.0004
SLC25A6	293	10.86	11.57	-0.71	0.00043
**FGR**	2268	9.39	10.53	-1.13	0.00059
NUP210	23225	6.66	7.48	-0.81	0.00068
RHOF	54509	5.82	6.94	-1.12	0.00068
PBX2	5089	7.06	7.64	-0.58	0.0007
CCND3	896	8.81	9.53	-0.71	0.0007
**CFP**	5199	6.44	7.77	-1.34	0.00081
ICAM3	3385	6.56	7.76	-1.2	0.00093
EIF4EBP2	1979	7.83	8.42	-0.59	0.00104
**FYN**	2534	6.14	7.22	-1.07	0.00109
CD244	51744	5.14	6.09	-0.95	0.00112
RAB11FIP4	84440	5.73	6.37	-0.65	0.00119
SMAD3	4088	5.91	6.56	-0.65	0.00119
**CYTIP**	9595	9.11	9.94	-0.83	0.00126
EIF3E	3646	8.19	8.71	-0.51	0.00149
RPL15	6138	8.28	8.79	-0.5	0.00166
LIMD2	80774	5.99	6.5	-0.51	0.00166
ZBTB18	10472	6.57	7.21	-0.65	0.0017
**EMR3**	84658	5.51	6.98	-1.47	0.0017
CSNK1G2	1455	7.07	7.59	-0.52	0.00183
GSTP1	2950	9.74	10.3	-0.56	0.00196
BCL11A	53335	5.33	6.54	-1.21	0.00197
FCHO1	23149	6.1	6.62	-0.52	0.00199
ATP8A1	10396	5.96	6.76	-0.8	0.00199
RPL31	6160	8	8.68	-0.68	0.00207
UPK3A	7380	5.03	5.59	-0.56	0.00223
ITGA4	3676	5.84	6.78	-0.94	0.00223
GLTSCR2	29997	8.11	8.66	-0.55	0.00224
LSP1	4046	7.96	8.87	-0.91	0.00228
SEZ6L	23544	4.82	5.4	-0.58	0.0025
TSPAN32	10077	5.87	6.66	-0.78	0.00254
EIF3G	8666	9.65	10.18	-0.53	0.00267
KIAA0922	23240	7.48	8.29	-0.81	0.00288
JAK1	3716	8.75	9.34	-0.59	0.00289
**NEDD9**	4739	6.77	7.65	-0.88	0.00298
ACAA1	30	8.38	8.86	-0.48	0.00316
P2RY10	27334	4.45	5.22	-0.77	0.00319
SIGIRR	59307	6.23	6.73	-0.51	0.00327
**TES**	26136	8	8.75	-0.75	0.0033
RPS23	6228	7.21	7.77	-0.56	0.00366
RPS16	6217	9.18	9.64	-0.46	0.00375
MED28	80306	7.76	8.26	-0.49	0.00375
**CD48**	962	8.97	9.82	-0.85	0.00375
RAC2	5880	8.37	9.26	-0.88	0.00375

CD101	9398	6.47	7.63	-1.16	0.00375
SF3A1	10291	8.16	8.68	-0.53	0.00392
USP3	9960	9.49	10.02	-0.53	0.00421
SPN	6693	6.63	7.43	-0.81	0.00424
S1PR4	8698	6.86	7.41	-0.55	0.00434
**LYST**	1130	7.27	8.34	-1.07	0.00459
RAB11FIP1	80223	7.96	8.58	-0.62	0.00463
CNN2	1265	8.35	8.93	-0.58	0.0047
POLR1D	51082	9.04	9.51	-0.47	0.00475
C15orf39	56905	6.58	7.21	-0.63	0.00475
TRAF3IP3	80342	5.66	6.48	-0.83	0.00504
MST4	51765	6.54	7.31	-0.77	0.00545
RNF41	10193	6.25	6.73	-0.48	0.00547
FCER1A	2205	4.97	6.36	-1.39	0.00575
RNF24	11237	6.52	7.18	-0.66	0.00582
WAC	51322	8.02	8.49	-0.47	0.0059
BID	637	8.81	9.36	-0.56	0.006
FLT3	2322	5.43	6.36	-0.93	0.00616
MTMR14	64419	7.39	7.85	-0.46	0.00625
PRKCE	5581	5.48	5.98	-0.49	0.00625
ZNF652	22834	5.99	6.85	-0.85	0.00625
CNOT8	9337	8.02	8.62	-0.6	0.00637
PTP4A2	8073	9.97	10.51	-0.54	0.0066
FLOT2	2319	7.64	8.29	-0.65	0.00663
CD11C	3687	9.01	9.9	-0.89	0.00663
NACA	4666	8.7	9.18	-0.48	0.00663
APOBR	55911	7.27	7.91	-0.63	0.00681
CA5B	11238	5.6	6.09	-0.49	0.00683
**CD52**	1043	10.72	11.48	-0.76	0.00683
KLF12	11278	5.48	6.26	-0.78	0.00683
FAM117A	81558	6.35	6.91	-0.56	0.00687
INSR	3643	6.55	7.2	-0.65	0.00687
NLRP1	22861	5.88	6.62	-0.74	0.00693
CS	1431	9.84	10.19	-0.35	0.00711
PELI2	57161	6.19	6.87	-0.68	0.00739
APAF1	317	7.06	7.65	-0.6	0.00741
MAP4K1	11184	6.29	7.04	-0.76	0.00741
GALNT3	2591	4.82	5.39	-0.57	0.00741
RASSF2	9770	7.71	8.46	-0.75	0.0076
EIF4B	1975	7.63	8.4	-0.77	0.0076
CPPED1	55313	7.23	8.05	-0.82	0.0076
**CCDC88C**	440193	4.64	5.48	-0.84	0.0076
CD1C	911	6.2	7.52	-1.31	0.0076
PLP2	5355	9.74	10.44	-0.7	0.00777
PPM1F	9647	7.17	7.63	-0.46	0.00781
VPS51	738	8.27	8.71	-0.44	0.00818
RBL2	5934	6.79	7.34	-0.55	0.00818
IL16	3603	6.91	7.34	-0.44	0.00912
EIF3M	10480	9.05	9.61	-0.56	0.00925
ITPK1	3705	8.55	8.98	-0.43	0.00926
EIF3H	8667	7.3	7.86	-0.55	0.00926

MEFV	4210	4.72	5.35	-0.63	0.00931
PRKCB	5579	6.61	7.54	-0.93	0.00949
ZBTB11	27107	7.77	8.24	-0.47	0.00966
UNC119	9094	7.54	7.98	-0.44	0.00975
PGLS	25796	8.03	8.59	-0.55	0.00975
STK17B	9262	7.68	8.45	-0.78	0.00975
ZDHHC18	84243	7.58	8.08	-0.5	0.00991
OXA1L	5018	9.68	10.07	-0.39	0.00992
GSE1	23199	7.04	7.61	-0.57	0.01
DNAJC4	3338	6.99	7.51	-0.53	0.01007
TKT	7086	9.12	9.79	-0.67	0.01007
GMFG	9535	10.23	10.68	-0.46	0.01017
RPL36	25873	9.89	10.32	-0.43	0.01024
TOB1	10140	8.36	9.23	-0.87	0.01035
RPS27	6232	8.08	8.48	-0.41	0.0104
NIN	51199	7.34	7.81	-0.46	0.0104
IMPA2	3613	6.92	7.57	-0.64	0.0104
**CDC42EP3**	10602	7.41	8.31	-0.9	0.0104
CDKN1B	1027	9.32	9.83	-0.51	0.01092
CAT	847	8.61	9.31	-0.7	0.01099
ESYT1	23344	9.09	9.58	-0.48	0.01101
FDFT1	2222	8.65	9.2	-0.55	0.01123
DGKE	8526	4.68	5.18	-0.5	0.01138
FBL	2091	9.28	9.73	-0.45	0.01139
AGTPBP1	23287	7.8	8.33	-0.53	0.01162
ATP2A3	489	5.71	6.32	-0.61	0.0118
CORO1A	11151	8.09	8.87	-0.78	0.01185
9月-09	10801	6	6.57	-0.57	0.01194
SIK3	23387	6.15	6.75	-0.59	0.01194
KAT6A	7994	6.87	7.28	-0.41	0.01196
RSL1D1	26156	7.61	8.09	-0.48	0.012
C1RL	51279	6.47	7.17	-0.69	0.012
GABBR1	2550	5.84	6.38	-0.54	0.012
**CAPN2**	824	9.86	10.6	-0.74	0.01213
XYLT1	64131	6.58	7.39	-0.82	0.01219
NDUFA10	4705	8.51	8.94	-0.43	0.01226
MPHOSPH9	10198	6.25	6.74	-0.5	0.01241
NDST1	3340	6.13	6.6	-0.47	0.0126
C20orf27	54976	6.14	6.75	-0.61	0.0132
PTPN6	5777	9.75	10.27	-0.52	0.01332
NAP1L1	4673	9.09	9.66	-0.57	0.01333
PSIP1	11168	6.76	7.39	-0.63	0.01348
MAN2A2	4122	6.98	7.41	-0.43	0.01348
ACAP1	9744	5.72	6.32	-0.6	0.01348
ZNF467	168544	5.78	6.22	-0.43	0.01351
SLC9A3R1	9368	7.68	8.15	-0.46	0.01354
CERK	64781	7.72	8.18	-0.46	0.01361
PDE4A	5141	6.02	6.65	-0.63	0.01363
RBM3	5935	6.88	7.39	-0.51	0.01376
RFX7	64864	7.23	7.67	-0.44	0.01383
ELF4	2000	8.01	8.46	-0.45	0.01404

CDKN2D	1032	5.96	6.42	-0.46	0.01414
ROGDI	79641	7	7.55	-0.55	0.01425
**FLNA**	2316	8.08	8.82	-0.74	0.01475
WDR48	57599	7.3	7.76	-0.47	0.01514
FAM60A	58516	6.69	7.26	-0.56	0.01514
CASP1	834	8.6	9.42	-0.83	0.01539
AIP	9049	7.26	7.69	-0.43	0.01541
TESC	54997	5.29	6.02	-0.73	0.01545
LCP1	3936	11.4	11.85	-0.45	0.01549
CYLD	1540	8.07	8.54	-0.47	0.01549
RARA	5914	6.19	6.9	-0.71	0.01549
NEK9	91754	7.55	7.92	-0.38	0.01551
VCL	7414	8.14	8.85	-0.71	0.01552
ABCA7	10347	6.27	6.69	-0.42	0.0158
IL18R1	8809	4.28	4.92	-0.64	0.01618
ADRBK2	157	8.19	8.64	-0.46	0.01621
RUNX3	864	7.46	8.11	-0.65	0.01656
CCNG1	900	9	9.48	-0.47	0.01661
SCRN1	9805	7.57	8.2	-0.62	0.01661
AHCYL2	23382	6.29	6.7	-0.41	0.0167
PHF17	79960	5.83	6.32	-0.48	0.01672
SETD2	29072	6.14	6.55	-0.41	0.01732
PCYOX1L	78991	7.21	7.7	-0.48	0.01765
AES	166	7.76	8.22	-0.46	0.01788
**PLAC8**	51316	7.56	8.84	-1.28	0.01792
SULT1B1	27284	5.2	5.7	-0.5	0.01799
RIN3	79890	7.1	7.8	-0.7	0.01799
RPS21	6227	8.66	9.04	-0.37	0.01818
RAP1GAP2	23108	6.44	7.05	-0.61	0.01826
DHPS	1725	7.38	7.8	-0.42	0.01832
PITPNM1	9600	6.47	6.91	-0.44	0.01837
VRK1	7443	7.18	7.63	-0.45	0.0186
ADAP1	11033	7.48	7.96	-0.48	0.0187
ATP11B	23200	6.42	6.91	-0.49	0.01883
TUBA4A	7277	7.17	7.76	-0.58	0.01891
WDR82	80335	9.5	9.87	-0.37	0.01893
FRAT2	23401	7.63	8.08	-0.45	0.01898
ZFAND1	79752	7.27	7.75	-0.48	0.01902
LRMP	4033	7.08	7.76	-0.68	0.01971
CPSF6	11052	6.52	6.97	-0.45	0.01987
CD207	50489	4.71	5.56	-0.85	0.01987
C11orf21	29125	5.58	6.4	-0.82	0.01998
SRRM1	10250	8.61	8.93	-0.32	0.02036
BRD3	8019	7.75	8.11	-0.36	0.02036
SERTAD2	9792	9.21	9.6	-0.38	0.02036
STAG3L4	64940	6.24	6.69	-0.44	0.02036
DHTKD1	55526	6.23	6.7	-0.47	0.02036
NONO	4841	8.29	8.69	-0.4	0.02066
TBL1X	6907	6.31	7.09	-0.78	0.02072
EIF2S3	1968	7.6	8.33	-0.72	0.02081
LRBA	987	6.61	7.11	-0.5	0.02107

VAMP2	6844	6.94	7.38	-0.43	0.02115
MKNK2	2872	8.6	9.08	-0.48	0.02128
STAG2	10735	7.95	8.44	-0.48	0.0214
FMNL1	752	7.5	8.02	-0.53	0.0214
ELF2	1998	6.53	6.98	-0.44	0.02167
SETBP1	26040	5.3	5.74	-0.45	0.02167
MYO1F	4542	9.27	9.75	-0.48	0.02167
SPATA6	54558	5.31	5.84	-0.54	0.02225
DAPP1	27071	7.76	8.38	-0.63	0.02236
IDH3A	3419	8.05	8.51	-0.46	0.02244
TMEM104	54868	6.98	7.35	-0.37	0.02255
MBNL3	55796	7.27	7.75	-0.48	0.02255
CCNI	10983	8.1	8.64	-0.54	0.02255
ZNF318	24149	6.89	7.28	-0.39	0.02257
RASGRP2	10235	5.83	6.4	-0.57	0.02257
PYCARD	29108	9.79	10.25	-0.46	0.02272
BACH2	60468	4.11	4.46	-0.35	0.02296
STAT5B	6777	6.81	7.19	-0.38	0.02348
C6orf48	50854	8.36	8.91	-0.55	0.0235
ARF5	381	7.99	8.36	-0.37	0.02353
RPS27A	6233	8.58	8.97	-0.39	0.02372
MEF2D	4209	6.9	7.3	-0.4	0.02378
MEX3C	51320	6.84	7.35	-0.52	0.02378
CELF2	10659	8.11	8.69	-0.57	0.02387
GDI2	2665	9.76	10.23	-0.47	0.02409
OSBPL8	114882	7.37	8.09	-0.72	0.02484
GID8	54994	8.29	8.65	-0.36	0.02508
MAP2K3	5606	7.4	7.97	-0.57	0.02535
IPCEF1	26034	5.85	6.52	-0.66	0.02576
ARAF	369	7.87	8.23	-0.36	0.02606
CCDC69	26112	6.21	6.83	-0.61	0.02606
FRY	10129	6.65	7.45	-0.81	0.02606
SNRK	54861	8.11	8.56	-0.46	0.02618
SVIL	6840	6.72	7.43	-0.71	0.02618
SPINT1	6692	7.23	7.74	-0.51	0.02656
AFF3	3899	4.84	5.46	-0.62	0.02656
ARHGEF6	9459	9.37	9.79	-0.42	0.02686
CREBBP	1387	7.33	7.67	-0.34	0.02705
VEZF1	7716	8.31	8.75	-0.44	0.02736
DIAPH1	1729	8.1	8.56	-0.46	0.02736
CHST2	9435	6.02	6.57	-0.55	0.02736
CDC40	51362	6.68	7.1	-0.42	0.02849
RPL14	9045	8.91	9.39	-0.48	0.02868
RMND5A	64795	6.93	7.34	-0.41	0.02929
CIDEB	27141	6.96	7.42	-0.46	0.02941
LIMD1	8994	6.89	7.33	-0.44	0.03059
CHAF1A	10036	5.81	6.14	-0.33	0.03059
TP53	7157	6.67	7.15	-0.48	0.03059
BAG1	573	7.88	8.28	-0.4	0.03104
INPP5F	22876	5.84	6.35	-0.51	0.03104
TRAPPC6A	79090	7.42	7.81	-0.38	0.03112

ANP32A	8125	8.95	9.35	-0.4	0.03123
SLC12A6	9990	6.86	7.43	-0.57	0.03215
PMS2P1	5379	6.89	7.3	-0.41	0.03272
RNF126	55658	6.92	7.3	-0.37	0.03289
BPTF	2186	5.81	6.14	-0.34	0.03306
SELL	6402	6.73	7.49	-0.76	0.03327
PNRC2	55629	8.69	9.05	-0.36	0.03354
VIPR1	7433	5.58	6.08	-0.5	0.03366
TMEM8B	51754	5.77	6.19	-0.42	0.03369
SLC25A40	55972	8.48	8.96	-0.48	0.03369
NADSYN1	55191	7.96	8.31	-0.35	0.03371
PPP3CA	5530	8.44	8.9	-0.47	0.03383
NCF2	4688	10.88	11.37	-0.48	0.03383
ZNF592	9640	7.31	7.65	-0.34	0.03388
TMEM66	51669	7.54	7.91	-0.37	0.0344
IRF4	3662	5.92	6.57	-0.65	0.03444
LSM6	11157	8.82	9.2	-0.38	0.03475
POLR1E	64425	5.27	5.6	-0.34	0.03482
URI1	8725	7.23	7.63	-0.4	0.03482
TNKS2	80351	7.94	8.39	-0.46	0.03482
STK24	8428	8.35	8.69	-0.34	0.03482
LIG3	3980	5.93	6.26	-0.33	0.03497
HIVEP1	3096	6.93	7.49	-0.56	0.03511
FAM168B	130074	7.59	8	-0.41	0.03547
SLC38A1	81539	5.58	6.49	-0.92	0.03584
SPIB	6689	5.16	5.66	-0.5	0.03589
SLCO3A1	28232	7.28	7.88	-0.6	0.03598
CYFIP2	26999	6.5	7.21	-0.7	0.03598
AMPD2	271	7.62	8.22	-0.6	0.0363
DCLRE1C	64421	6.68	7.08	-0.4	0.03652
NFE2	4778	4.43	4.95	-0.52	0.03652
CARS2	79587	8.43	8.78	-0.35	0.03687
G3BP2	9908	8.25	8.62	-0.37	0.03738
PPP6C	5537	8.9	9.26	-0.36	0.03765
SLC25A28	81894	7.5	7.91	-0.41	0.03765
RSL24D1	51187	9.06	9.46	-0.41	0.03801
MTMR1	8776	7.46	8	-0.54	0.03801
RPS6KA3	6197	9	9.41	-0.41	0.03837
NCOA1	8648	8.07	8.44	-0.37	0.03845
EPB41	2035	6.1	6.49	-0.4	0.03845
RPL23A	6147	9.34	9.79	-0.45	0.03845
9月-06	23157	7.3	7.79	-0.48	0.03845
LST1	7940	9.87	10.42	-0.55	0.03845
PACS1	55690	6.94	7.27	-0.33	0.0385
LNPEP	4012	7.13	7.65	-0.53	0.03871
RCOR1	23186	7.83	8.17	-0.34	0.03919
OBFC1	79991	6.69	7.02	-0.34	0.03919
CASP2	835	5.73	6.14	-0.41	0.03919
USP48	84196	6.51	6.85	-0.34	0.03928
IFT20	90410	8.71	9.13	-0.42	0.03928
SCAF4	57466	6.47	6.9	-0.43	0.03936

CBX7	23492	6.92	7.28	-0.36	0.03955
RGS14	10636	5.77	6.15	-0.38	0.03997
NAAA	27163	7.98	8.49	-0.52	0.04045
CHP1	11261	7.62	8.04	-0.42	0.04045
SLC25A38	54977	7.26	7.59	-0.33	0.04047
MKL1	57591	7.13	7.5	-0.37	0.04047
LSM7	51690	8.42	8.79	-0.38	0.04047
EFHD2	79180	9.56	10.01	-0.46	0.04062
ZBTB33	10009	8.84	9.18	-0.34	0.04082
SH2D3C	10044	5.5	6.01	-0.51	0.04133
CD1E	913	4.58	5.72	-1.14	0.04144
C2orf49	79074	5.79	6.1	-0.31	0.04147
PDE6G	5148	6.41	6.77	-0.36	0.04155
CRBN	51185	8.36	8.75	-0.4	0.04155
CLEC4A	50856	7.78	8.56	-0.77	0.04155
**IRAK3**	11213	7.18	8.02	-0.84	0.04155
NOTCH2	4853	8.19	8.73	-0.54	0.04161
KCTD15	79047	4.8	5.28	-0.47	0.04203
SMARCC1	6599	7.39	7.75	-0.35	0.04211
BCL2L13	23786	6.48	6.82	-0.35	0.04214
KDM4B	23030	7.02	7.38	-0.36	0.04241
SLC25A12	8604	6.28	6.62	-0.34	0.04242
ERLIN2	11160	6.42	6.85	-0.44	0.04242
KLF13	51621	7.21	7.74	-0.54	0.04242
CD37	951	9.15	9.74	-0.59	0.04242
U2SURP	23350	6.31	6.79	-0.48	0.04251
MAML3	55534	5.91	6.43	-0.52	0.04256
ZNF281	23528	7.48	7.86	-0.39	0.04402
RFTN1	23180	8.65	9.08	-0.43	0.04425
C21orf91	54149	6.51	6.98	-0.47	0.04425
ANAPC15	25906	7.64	7.99	-0.35	0.04443
UQCRB	7381	6.38	6.75	-0.37	0.04443
PLCB2	5330	8.01	8.41	-0.4	0.04443
AKIRIN1	79647	7.91	8.35	-0.44	0.04443
STK4	6789	7.23	7.79	-0.56	0.04443
METTL9	51108	8.88	9.4	-0.52	0.04447
PHF20	51230	7.39	7.84	-0.45	0.04452
IPO5	3843	7.6	8.06	-0.46	0.04487
CBX4	8535	7.15	7.56	-0.41	0.04535
RSBN1	54665	7.42	7.85	-0.43	0.04573
SLC1A5	6510	7.39	7.8	-0.4	0.04585
LANCL1	10314	7.85	8.21	-0.36	0.04716
ATPAF2	91647	6.47	6.81	-0.33	0.04729
HNRNPUL1	11100	7.95	8.31	-0.36	0.04729
ZMIZ1	57178	8.79	9.11	-0.32	0.04753
SCN9A	6335	3.97	4.4	-0.44	0.04753
OGT	8473	6.57	7.18	-0.61	0.04768
KLHDC2	23588	8.3	8.68	-0.39	0.04794
TMPO	7112	7.13	7.65	-0.52	0.04844
EVI2B	2124	11.04	11.39	-0.36	0.04884
KDM5A	5927	6.64	7.03	-0.39	0.04884

UQCRC2	7385	6.85	7.21	-0.36	0.04892
FXYD5	53827	9.88	10.31	-0.43	0.04892
CBX6	23466	7.35	7.72	-0.38	0.04939
DDX46	9879	7.73	8.06	-0.33	0.0495
ADRBK1	156	7.28	7.7	-0.42	0.04983
